# Convergence of BMI1 and CHD7 on ERK Signaling in Medulloblastoma

**DOI:** 10.1016/j.celrep.2017.11.021

**Published:** 2017-12-05

**Authors:** Sara Badodi, Adrian Dubuc, Xinyu Zhang, Gabriel Rosser, Mariane Da Cunha Jaeger, Michelle M. Kameda-Smith, Anca Sorana Morrissy, Paul Guilhamon, Philipp Suetterlin, Xiao-Nan Li, Loredana Guglielmi, Ashirwad Merve, Hamza Farooq, Mathieu Lupien, Sheila K. Singh, M. Albert Basson, Michael D. Taylor, Silvia Marino

**Affiliations:** 1 Blizard Institute, Barts and The London School of Medicine and Dentistry, Queen Mary University of London, 4 Newark Street, London E1 2AT, UK; 2 Program in Developmental & Stem Cell Biology, The Hospital for Sick Children, 101 College Street, TMDT-11-401M, Toronto, ON M5G 1L7, Canada; 3 Pediatric Neurosurgery, Department of Surgery, McMaster Children’s Hospital and McMaster Stem Cell & Cancer Research Institute, MDCL 5027, 1280 Main Street West, Hamilton, ON L8S 4K1, Canada; 4 Princess Margaret Cancer Centre, University Health Network, Toronto, ON, Canada; 5 Department of Medical Biophysics, University of Toronto, Toronto, ON, Canada; 6 Ontario Institute for Cancer Research, Toronto, ON, Canada; 7 Department of Craniofacial Development and Stem Cell Biology, King’s College London, Floor 27, Guy’s Hospital Tower Wing, London SE1 9RT, UK; 8 Texas Children’s Cancer Centre, Texas Children’s Hospital, Baylor College of Medicine, 6621 Fannin Street, MC-3-3320, Houston, TX 77479, USA

**Keywords:** BMI1, CHD7, DUSP4, ERK, medulloblastoma, PcG genes, mouse models, epigenetics, chromatin

## Abstract

We describe molecular convergence between BMI1 and CHD7 in the initiation of medulloblastoma. Identified in a functional genomic screen in mouse models, a BMI1^High^;CHD7^Low^ expression signature within medulloblastoma characterizes patients with poor overall survival. We show that BMI1-mediated repression of the ERK1/2 pathway leads to increased proliferation and tumor burden in primary human MB cells and in a xenograft model, respectively. We provide evidence that repression of the ERK inhibitor DUSP4 by BMI1 is dependent on a more accessible chromatin configuration in G4 MB cells with low CHD7 expression. These findings extend current knowledge of the role of BMI1 and CHD7 in medulloblastoma pathogenesis, and they raise the possibility that pharmacological targeting of BMI1 or ERK may be particularly indicated in a subgroup of MB with low expression levels of CHD7.

## Highlights

• CHD7 inactivation induces MB in a forward genetic screen in Bmi1-overexpressing mice
• A BMI1^High^;CHD7^Low^ signature is found in MB patients with poor overall survival
• CHD7^Low^ favors chromatin accessibility at PcG target genes
• ERK overactivity increases proliferation and tumor burden in BMI1^High^;CHD7^Low^ MB

## Graphical Abstract

**Figure:**
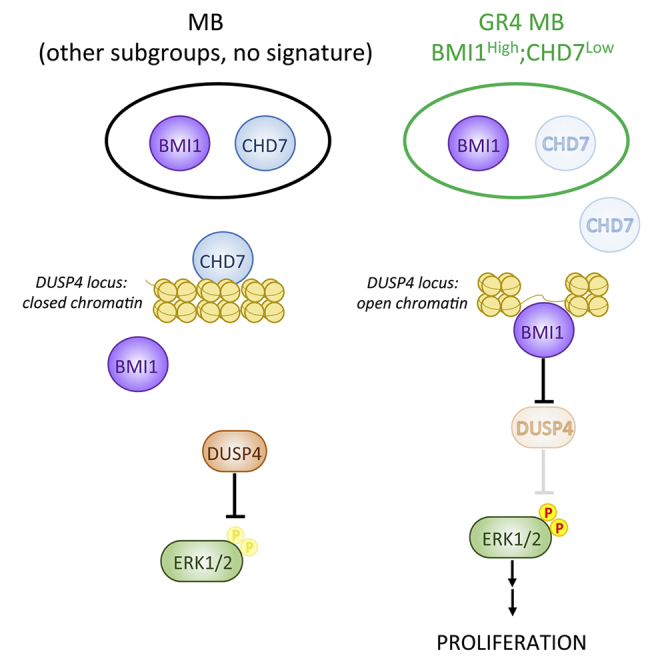

## Teaser

Badodi et al. find convergence of the chromatin modifiers BMI1 and CHD7 in medulloblastoma pathogenesis, and they show that this pathway regulates tumor proliferation and growth via ERK signaling.

## Introduction

Medulloblastomas (MBs) are the most common malignant brain tumors of childhood. Current standard of care consists of multimodal therapy (surgery with radio- and/or chemotherapy) that does not take into account the specific molecular mechanisms driving tumor growth. Despite a good overall survival rate, long-term survivors face significant treatment-related morbidity and reduced quality of life. Risk stratification of patients based on molecular subtyping and targeted therapies are essential to reduce secondary effects and improve overall prognosis.

Gene expression profiling of large cohorts of MBs has dissected this historically monomorphous tumor entity into four distinct molecular subgroups (WNT, SHH, group 3, and group 4) with divergent prognoses and responses to therapy (Taylor et al., 2012). Recently, heterogeneity within these subgroups has been reduced by two independent studies that have defined additional subtypes (Cavalli et al., 2017, Schwalbe et al., 2017). While WNT, SHH, and group (G)3 MBs are well characterized molecularly and have been shown to arise from topographically distinct neural progenitor cells (reviewed in Leto et al., 2016), little is known about the molecular and cellular mechanisms underpinning the formation of G4 MB, although it is the most frequent (Ramaswamy et al., 2016).

Oncogenic events affecting chromatin-modifying genes have been described across the subgroups (Dubuc et al., 2013), although different modifications are found in the specific subgroups and their frequency varies (Jones et al., 2013). Inactivating mutations in the histone demethylase *KDM6A* and loss-of-function mutations in the Trithorax gene *MLL3* (Northcott et al., 2012) as well as elevated expression of the Polycomb proteins EZH2 (Robinson et al., 2012) and BMI1 (Behesti et al., 2013) were identified particularly in G4 MBs, raising the possibility that increased Polycomb repression is an important step in the pathogenesis of MBs and in particular of G4 tumors.

The Polycomb group protein BMI1 is a potent inducer of neural stem cell self-renewal and neural progenitor cell proliferation during development and in adult tissue homeostasis (Leung et al., 2004). Numerous studies have demonstrated that BMI1, which is upregulated in a variety of cancers, has a positive correlation with clinical grade/stage and poor prognosis (Glinsky et al., 2005). BMI1 is overexpressed across all MB subgroups, with the highest levels of expression detected in G4 tumors, followed by G3, SHH, and WNT (Behesti et al., 2013). The growth of G4 MBs is dependent on BMI1 expression, as BMI1 knockdown results in reduced tumor growth and invasion in a xenograft model (Merve et al., 2014). Also SHH MB growth is dependent on BMI1 expression, as demonstrated by experimental genetic approaches where crossing a mouse model of SHH MB onto a *Bmi1*^−/−^ background neutralized tumor formation (Michael et al., 2008). Intriguingly, we recently found that overexpression of Bmi1 alone in cerebellar granule cell progenitors (GCPs), a cell of origin of MB, is not sufficient to induce MB formation (Behesti et al., 2013). As overexpression of Bmi1 driven by the pan-neural Cre line Nestin-Cre did not yield MB formation (Yadirgi et al., 2011), it is conceivable that additional oncogenic events are required to collaborate with BMI1 to induce MB formation.

Here we used a Sleeping Beauty forward genetic approach in the mouse to identify oncogenic events collaborating with Bmi1 overexpression to induce MB formation. We identify a molecular convergence between Bmi1 and Chd7, an ATP-dependent chromatin remodeler that fine-tunes developmental gene expression through modulating chromatin structure (reviewed in Basson and van Ravenswaaij-Arts, 2015). Importantly, somatic mutations in *CHD7* have been described in G4 MBs (Robinson et al., 2012), although their tumorigenicity *in vivo* has not been proven. Here we show that CHD7 controls the proliferation of G4 MB cells and its silencing renders them susceptible to BMI1-mediated ERK1/2-induced proliferation control, raising the possibility that BMI1 and/or ERK may be promising druggable targets in this MB subgroup.

## Results

### A Forward Genetic Screen Identifies *Chd7* as a Frequent Insertion Site in MB Arising in Mice Overexpressing Bmi1 in Cerebellar Progenitors

**Figure 1:**
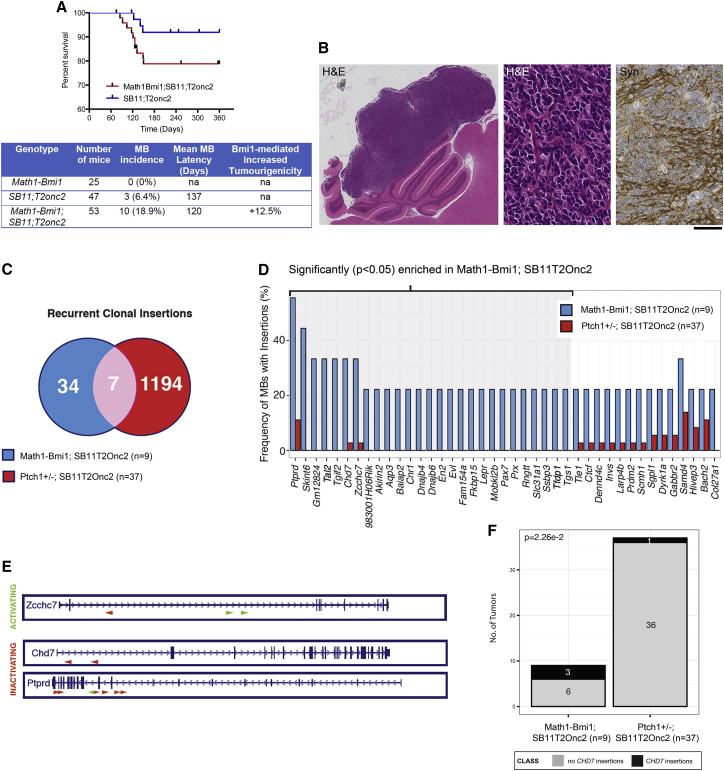
Identification of Oncogenic Events Cooperating with Bmi1 to Promote MB Development Using the Sleeping Beauty Transposon System (A) Kaplan-Meier survival analysis and summary of animal models used to assess the contributions of Bmi1 to MB development. The addition of Bmi1 overexpression to SB11;T2Onc2 led to increased MB formation. (B) Histology shows morphological (H&E) and immunohistochemical (Synaptophysin) features of MB in Math1-Bmi1;SB11;T2Onc2. (C) Comparison of recurrent Sleeping Beauty insertions identified in Math1-Bmi1;SB11;T2Onc2 with those identified in Ptch1^+/−^;SB11;T2Onc2 mice (Morrissy et al., 2016) highlighted *Chd7* as a recurrent clonal insertion. (D) Bar graph representation of recurrent Sleeping Beauty insertions in Math1-Bmi1;SB11;T2Onc2 versus Ptch^+/−^;SB11;T2Onc2 mice, highlighting events significantly (p < 0.05) enriched following Bmi1 overexpression. (E) Sleeping Beauty insertion maps for candidate genes identified in Math1-Bmi1;SB11;T2Onc2 mice, including *Zcchc7* (activating and inactivating insertions), *Chd7* (inactivating insertions), and *Ptprd* (inactivating and activating insertions). (F) Statistically significant enrichment of *Chd7* insertions identified in the Math1-Bmi1;SB11;T2Onc2 MB model versus the Ptch1^+/−^;SB11;T2Onc2 MB model. Scale bar represents 2 mm (B, left) and 125 μm (B, center and right). See also Figure S1.

The Sleeping Beauty (SB) transposon-based mutagenesis system, which uses a transposase and mutagenic transposon alleles to target mutagenesis to somatic cells of a given tissue in mice, was used to identify random mutations capable of driving tumor development. Mice overexpressing Bmi1 in glutamatergic progenitors (*Math1-Bmi1* transgenic line; Figures S1A and S1B) were crossed to *SB11;T2Onc2* (Copeland and Jenkins, 2010). Triple-mutant mice as well as double-mutant controls were generated and kept on tumor watch for 12 months. Ten of 53 *Math1Bmi1;SB11;T2Onc2* mice developed MBs (18.9% incidence), while only 3 MBs were detected in 47 *SB11;T2Onc2* mice (6.4% incidence), in keeping with an increased tumorigenicity in the triple-mutant mice of 12.5% (Figure 1A). All tumors had morphological and immunohistochemical characteristics of MBs, as assessed by H&E stain and synaptophysin immunostaining (Figure 1B), and histological features were similar irrespective of their genotype.

Next, the tumors were analyzed for common transposon insertion sites in genes (gCIS) to identify candidates that could cooperate with Bmi1 in MB development (Data S1). A total of 41 recurrent clonal insertions were identified in MBs originating in *Math1Bmi1;SB11;T2Onc2*, of which 7 were shared with a published dataset of Sleeping Beauty-induced insertion sites in MBs occurring in *Ptch1*^+/−^ mice (Morrissy et al., 2016, Wu et al., 2012) (Figure 1C). Among the 41 gCISs identified in *Math1Bmi1;SB11;T2Onc2*, 27 were found to be significantly (p < 0.05) enriched following Bmi1 overexpression and 3 of these were shared between the two Sleeping Beauty-induced MB models (Figure 1D). Sleeping Beauty insertions in Chromodomain helicase DNA-binding factor 7 (*Chd7*) were inactivating insertions and so were the majority of insertions in *Ptprd*, while the majority of insertions in *Zcchc7* were activating (Figure 1E). *Chd7* is an ATP-dependent chromatin remodeling factor (Bajpai et al., 2010); *Ptprd* is a member of the protein tyrosine phosphatase (PTP) family (Pulido et al., 1995); and *Zcchc7* is a component of a nucleolar TRAMP-like complex, an ATP-dependent exosome regulatory complex (Lubas et al., 2011). Notably, inactivating insertions in *Chd7* were significantly enriched in the *Math1Bmi1;SB11;T2Onc2* MB model (3/6) as compared to the *Ptch1*^+/−^*;SB11;T2Onc2* MB model (1/36) (p < 0.023) (Figure 1F). Immunostaining for Bmi1 and Chd7 in the Sleeping Beauty-induced MBs with *Chd7* insertions as compared to those without revealed similar and reduced expression, respectively (Figures S1C and S1D).

Taken together, our data suggest that Bmi1 and Chd7 cooperate to induce MB in a mouse model.

### A BMI1^High^;CHD7^Low^ Molecular Signature Identifies a Subgroup of G4 MBs with Reduced Overall Survival

**Figure 2:**
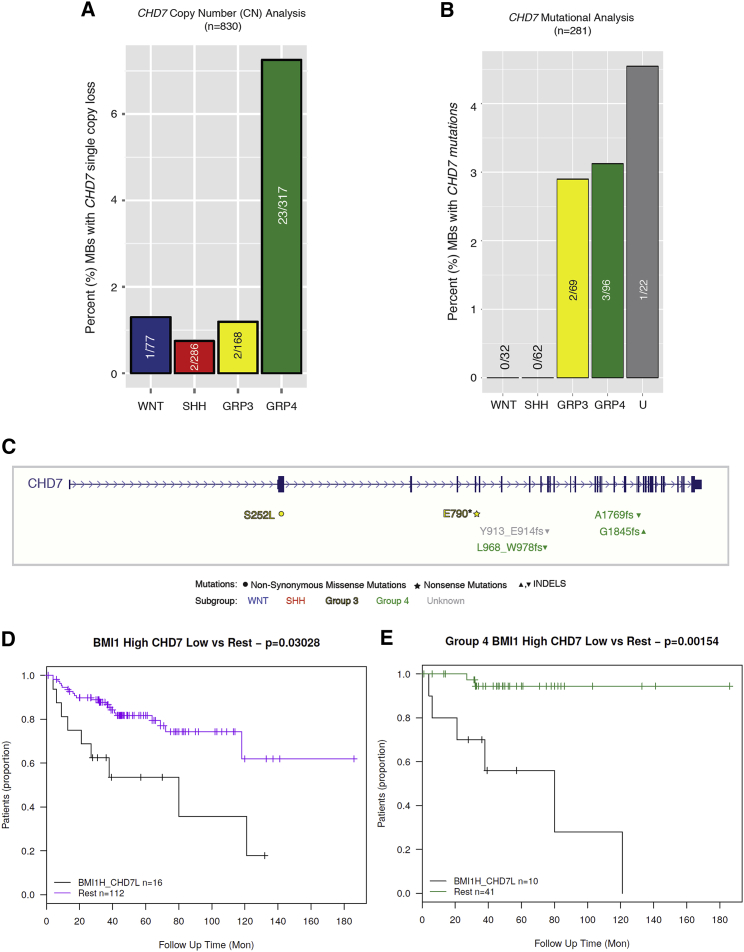
A BMI1^High^;CHD7^Low^ Signature in MB with Poor Prognosis (A) Graphical representation of the percentage of MB with *CHD7* single-copy loss across subgroups with enrichment in G4. (B) *CHD7* mutational analysis across molecular subgroups. (C) Graphical representation of the mutational inactivating events that affect *CHD7*. (D and E) Kaplan-Meier survival analysis demonstrates that tumors with a *BMI1*^High^;*CHD7*^Low^ signature have a significantly (p = 0.03028) reduced overall survival (D). When we examine this relationship in a subgroup-specific manner, the same trend (p = 0.00154) holds true for G4 MB (E). See also Figure S2.

Next, we screened primary human medulloblastoma samples for *CHD7* mutations. A collection of 830 primary human MBs profiled on Affymetrix SNP6.0 copy number arrays and subgrouped using Nanostring technology (Northcott et al., 2012) and 281 primary MBs subgrouped using next-generation sequencing (NGS) across three independent cohorts (Jones et al., 2012, Pugh et al., 2012, Robinson et al., 2012) were interrogated. Intriguingly, this analysis identified a molecular convergence on single-copy loss of *CHD7* via hemizygous deletion (Figure 2A) or hemizygous mutations (Figure 2B) in G4 MBs, while no significant enrichment for *CHD7* mutations was found in the SHH and WNT subgroups. A graphical representation of the mutational inactivating events that affect *CHD7* is shown in Figure 2C.

Next, we examined the subgroup-specific expression levels of *BMI1* and *CHD7* in a large transcriptomic dataset from primary human MBs (n = 187, run on Affymetrix HT-HG-U133A arrays) (Cho et al., 2011). While *CHD7* was most highly expressed in SHH MBs with relatively reduced expression in G3 and G4 MBs, the opposite patterns were observed for *BMI1* (Figures S2A and S2B). A Kaplan-Meier survival analysis demonstrated that tumors with a *BMI1*^High^ *CHD7*^Low^ expression profile had a significantly reduced overall survival as compared to MBs lacking this profile (Figure 2D; p = 0.03028). When we examined this relationship in a molecular subgroup-specific manner, a reduced overall survival was observed, particularly in G4 MBs with this signature (Figure 2E; p = 0.00154). No significant contribution to patients’ survival was observed when the relative contribution of *BMI1*^High^ or *CHD7*^Low^ was considered (Figure S2C).

These data support the notion that the Bmi1/Chd7 molecular convergence identified in mouse models reflects a potentially pathogenically relevant mechanism underpinning a subgroup of G4 MBs.

### CHD7 Controls Proliferation of G4 MB Cells *In Vitro* in a BMI1-Dependent Fashion

**Figure 3:**
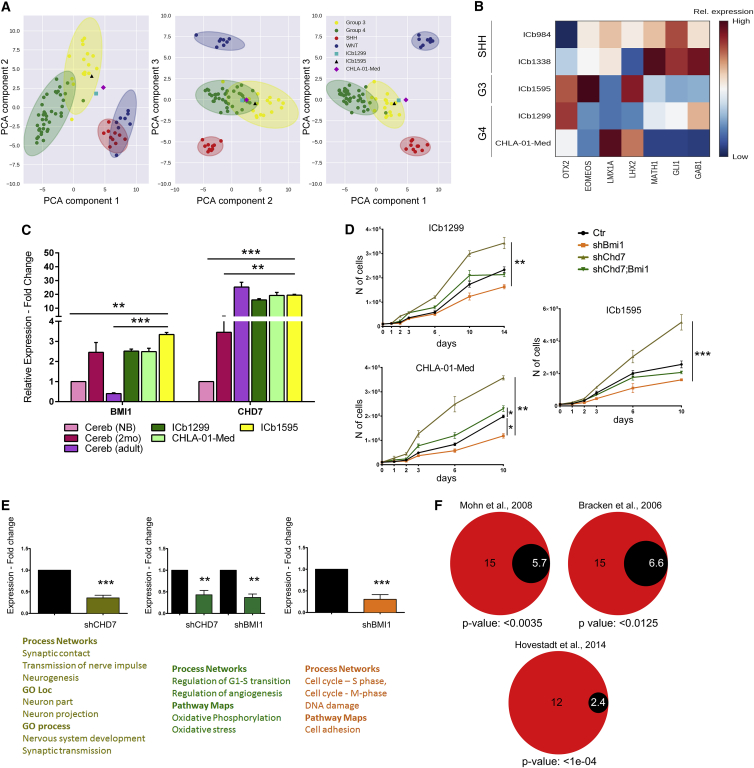
Increased Proliferation upon CHD7 Silencing Is BMI1-Dependent in Non-WNT, Non-SHH MB Patient-Derived Cell Models (A) Scatterplots of the first 3 principal components of the Robinson dataset (Robinson et al., 2012). Points are shaded based on their subgroup. Ellipses represent the 99% confidence intervals of each subgroup, computed using the covariance matrix of each subset. RNA-seq data of G4 (ICb1299 and CHLA-01-Med) and G3 primary MB cells were subjected to the same PCA transformation and are shown in cyan, magenta, and black respectively. (B) Heatmap representation of relative expression of selected MB markers in patient-derived cells (ICb1299 and CHLA-01-Med, G4; ICb1595, G3; and ICb1338 and ICb984, SHH). *OTX2*, *EOMEOS*, *LMX1A*, and *LHX2* are mainly expressed by G4 and G3 cells, while *GAB1*, *GLI1*, and *MATH1* are specifically expressed by SHH cells. (C) Expression of *BMI1* and *CHD7* in ICb1299, CHLA-01-Med, and ICb1595 patient-derived MB lines is comparable to their expression in the early postnatal cerebellum (*BMI1*) and adult cerebellum (*CHD7*). (D) Increased proliferation upon CHD7 silencing is BMI1 dependent (n = 3). (E) Pathway analysis reveals different processes and pathways affected by silencing CHD7 and BMI1 individually or concomitantly. (F) Diagram representing the fold enrichment of PcG target genes in the cohort of genes deregulated in CHD7-depleted cells, as compared to the expected values in murine NPCs (Mohn et al., 2008), in human fibroblasts (Bracken et al., 2006), and in human MBs (Hovestadt et al., 2014), as previously reported. Data are represented as mean ± SEM. See also Figure S3.

Patient-derived MB lines, some of which have been maintained as orthotopic xenografts (Zhao et al., 2012), were chosen to further dissect this molecular signature. First, we confirmed the subgroup affiliation by analyzing their gene espression against a classifier (Robinson et al., 2012). Principal-component analysis (PCA) of data obtained from RNA sequencing (RNA-seq) analysis confirmed that ICb1299, a line we have recently shown to be dependent on BMI1 for growth and intraparenchymal invasion in a xenograft model (Merve et al., 2014), and CHLA-01-Med belong to G4 with a significant overlap with G3, while ICb1595 belongs to G3 (Figure 3A). These lines were chosen for further analysis, and they were expanded and shortly maintained *in vitro* to perform functional studies. Hierarchical clustering of RNA-seq datasets of the cultured cells and the original datasets obtained from the patient’s tumor (Zhao et al., 2012) confirmed that, although some shift in gene expression did occur after *in vitro* culturing, they remained reasonably similar to the cells isolated from the xenografts and retained their subgroup affiliation (Figure S3A). Validation on independent cultures confirmed very high expression of *OTX2*, a transcription factor overexpressed in the majority of G4 MBs (Bunt et al., 2010) in all three lines, with concomitant high expression levels of the G3/G4 markers (Lin et al., 2016) *LMX1A* and *LHX2* in CHLA-01-Med and *EOMEOS* and *LHX2* in ICb1595 (Figure 3B). The expression of *MATH1*, *GLI1*, and *GAB1*, markers of granule cell progenitors and SHH MBs (Ayrault et al., 2010), was negligible in these lines compared to two patient-derived SHH MB lines, ICb984 and ICb1338 (Figure 3B).

Next, we compared the expression of *BMI1* and *CHD7* in these lines with human cerebellum, and we found similar expression levels for both genes in all MB lines and human cerebellum of early postnatal time points (newborn and 2 months of age). In contrast, the expression of *BMI1* was higher in all lines compared to adult cerebellum, while it was similar for CHD7 (Figure 3C). These findings verified that ICb1299 and CHLA-01-Med are suitable models to test the contribution of CHD7 to G4 MB pathogenesis in the context of BMI1 overexpression. Line ICb1595 was also analyzed further to dissect the specificity of the BMI1/CHD7 molecular convergence to G4 MBs.

Lentivirus-mediated CHD7 knockdown (Figure S3B) resulted in increased proliferation of all lines (Figure 3D), an effect that was BMI1 dependent as it was neutralized by concomitant BMI1 knockdown (Figure S3B; Figure 3D). Importantly, reconstitution of CHD7 expression in ICb1299 after silencing (Figure S3D) brought proliferation back to basal level (Figures S3E and S3F).

Together, these data indicate that CHD7 represses proliferation in G4 and G3 MB cells that overexpress BMI1.

### Genome-wide Expression Analysis Identifies CHD7 and BMI1 as Key Regulators of Neuronal Differentiation and Proliferation of G4 MB Primary Cells

To clarify the molecular mechanism mediating the synergistic effect between CHD7 and BMI1 in MB differentiation and proliferation, we carried out a genome-wide analysis of gene expression in the ICb1299 MB line. In particular, we set out to assess the impact of silencing CHD7 in a BMI1-overexpressing G4 MB primary cell line (shCHD7) compared with the same primary cell line treated with scrambled small hairpin RNA (shRNA) as a control (Ctr versus shCHD7). The impact of silencing BMI1 in shCHD7 cultures compared to silencing only CHD7 was also assessed (shCHD7 versus shCHD7;shBMI1), in addition to an evaluation of the impact of silencing BMI1 in the context of CHD7 expression (Ctr versus shBMI1). 360 genes were differentially expressed in Ctr versus shCHD7 (277 upregulated and 83 downregulated), 1,175 in shCHD7 versus shCHD7;shBMI1 (406 upregulated and 769 downregulated), and 584 in Ctr versus shBMI1 (224 upregulated and 360 downregulated) when thresholds of 0.05 for statistical significance and 0.6 for absolute log2 expression change were applied. First, we confirmed that no changes in the subgroup-defining marker expression were noted upon silencing CHD7 or BMI1 or both (Figure S3C). Pathway analysis carried out on the MetaCore platform highlighted different functions and pathways for these three comparisons. Interestingly, different terms and pathways were impacted in the different conditions tested. In the Ctr versus shCHD7 comparison, nervous system development, synaptic transmission (gene ontology [GO] process), neuron part, neuron projection (GO location), synaptic contact, transmission of nerve impulse, neurogenesis, and chromatin condensation in prometaphase (process networks) were significantly enriched. Regulation of G1-S transition, regulation of angiogenesis (process networks), oxidative phosphorylation, and oxidative stress (pathway maps) were highlighted for the comparison shCHD7 versus shCHD7;shBMI1; and cell adhesion (pathways maps) and cell cycle S phase and M phase as well as DNA damage were impacted in the Ctr versus shBMI1 comparison (Figure 3E).

Assessment of enrichment for Polycomb-group (PcG) targets among the differentially expressed (DE) genes in CHD7-depleted cells (using the statistical significant cutoff of p < 0.01) revealed that 15 of the 68 DE genes were previously reported to possess H3K27me3 on their promoters in mouse neural progenitor cells (Mohn et al., 2008), representing a 2.62-fold enrichment (p = 0.0035) (Figure 3F). Enrichments of 2.25-fold (p = 0.012) and 4.8-fold (p value < 1e−04) were found when the data were compared with the proportion of PcG target genes in human fibroblasts (Bracken et al., 2006) and in an MB dataset (Hovestadt et al., 2014), respectively (Figure 3F). These results support the notion that CHD7 functionally relates to the Polycomb complex in MB.

Taken together, these data suggest that the differentiation potential of MB G4 is controlled by CHD7 via direct or indirect interplay with genes repressed by the Polycomb complex in neural progenitors.

### Comparative Analysis with G4 MB Datasets Reveals Activation of ERK Pathway upon CHD7 Silencing via BMI1-Mediated Repression of DUSP4

**Figure 4:**
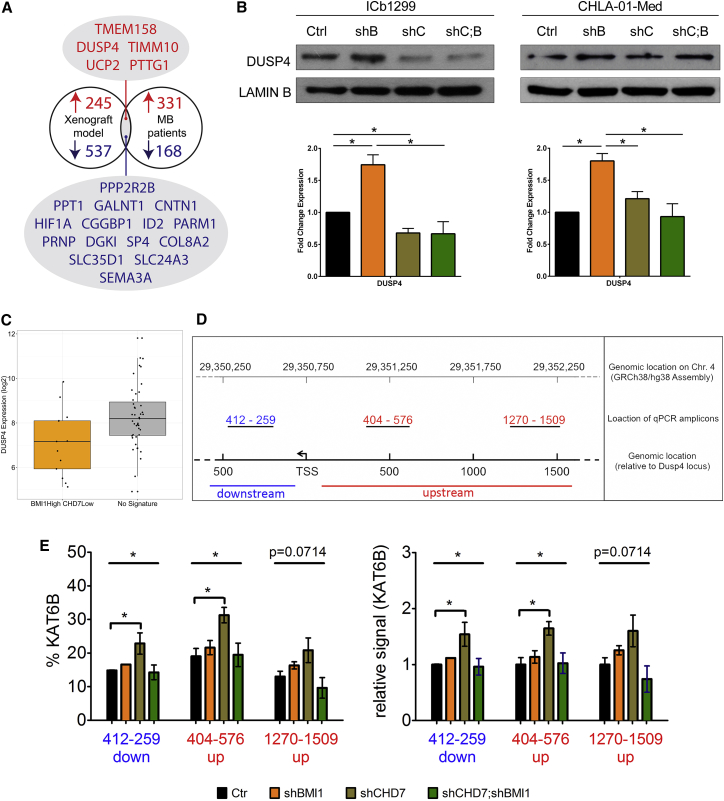
Molecular Convergence on Activation of ERK-Signaling Pathway upon CHD7 Silencing Is Mediated by the Repression of DUSP4 by BMI1 (A) Comparative analysis with human MB datasets identified conserved DE genes in G4 MB cells upon BMI1 knockdown in a CHD7-silenced context and in G4 MB with a BMI^High^;CHD7^Low^ signature. Venn diagram represents the analysis. (B) Western blot analysis (top) and quantification (bottom) show CHD7 dependency of BMI1-mediated repression of DUSP4 in G4 MB cells. Histograms show mean ± SEM (n = 3). (C) Boxplot showing lower expression of *DUSP4* in BMI1^High^;CHD7^Low^ G4 MB. (D) Assessment of DNA accessibility in the vicinity of the *DUSP4* TSS by assay for transposase-accessible chromatin (ATAC). Schematic representation of the promoter region of *DUSP4* with primer locations is shown. (E) Increased accessibility in CHD7-silenced cells is lost upon concomitant silencing of BMI1. Data are represented as mean ± SEM. See also Figure S4.

To identify the genes mediating the pro-proliferative effect of CHD7 silencing in a BMI1-overexpressing context and to ensure we were focusing on those relevant for MB patients, a comparative analysis with the expression data of the G4 MB subgroup of patients with a BMI1^High^;CHD7^Low^ signature (Figure 2E) was carried out. Five genes that were upregulated upon BMI1 knockdown in the shCHD7 cell model were also expressed at a lower level in the BMI1^High^;CHD7^Low^ patient group compared to the remaining patients, while 15 genes were downregulated in the shCHD7;shBMI1 cell model and expressed at higher levels in the BMI1^High^;CHD7^Low^ patient group (Figure 4A; Figure S4A). Pathway analysis carried out on the MetaCore platform for all 20 genes indicated ERK signal transduction as the most significant pathway affected (Figure S4B). A literature review identified *DUSP4*, *HIF-1α*, and *COL8A2* as being linked to ERK signaling, and analysis of a published dataset of chromatin immunoprecipitation sequencing (ChIP-seq) data for Bmi1 in neural stem cells and glioblastoma cells identified *Dusp4* as a direct Bmi1 target gene (Gargiulo et al., 2013). Moreover, several members of the DUSP gene family were upregulated in an RNA-seq dataset comparing neural stem cells lacking Bmi1 versus control in the mouse (Gargiulo et al., 2013). Upregulation of DUSP4 was confirmed in both G4 MB lines upon silencing of BMI1 (Figure 4B), and a lower expression level of *DUSP4* was confirmed in the G4 MB subgroup with a BMI1^High^;CHD7^Low^ signature (Figure 4C). Interestingly, no upregulation of DUSP4 expression was noted in the G3 MB line upon CHD7 silencing (Figure S4C). Next, we assessed chromatin accessibility in the vicinity of the *DUSP4* transcription start site (TSS) using the assay for transposase-accessible chromatin (ATAC) method (Buenrostro et al., 2013). Silencing of CHD7 increased DNA accessibility around the *DUSP4* TSS and the *DUSP4* promoter (Figures 4D and 4E), a configuration that was lost upon concomitant silencing of BMI1 (Figure 4E).

**Figure 5:**
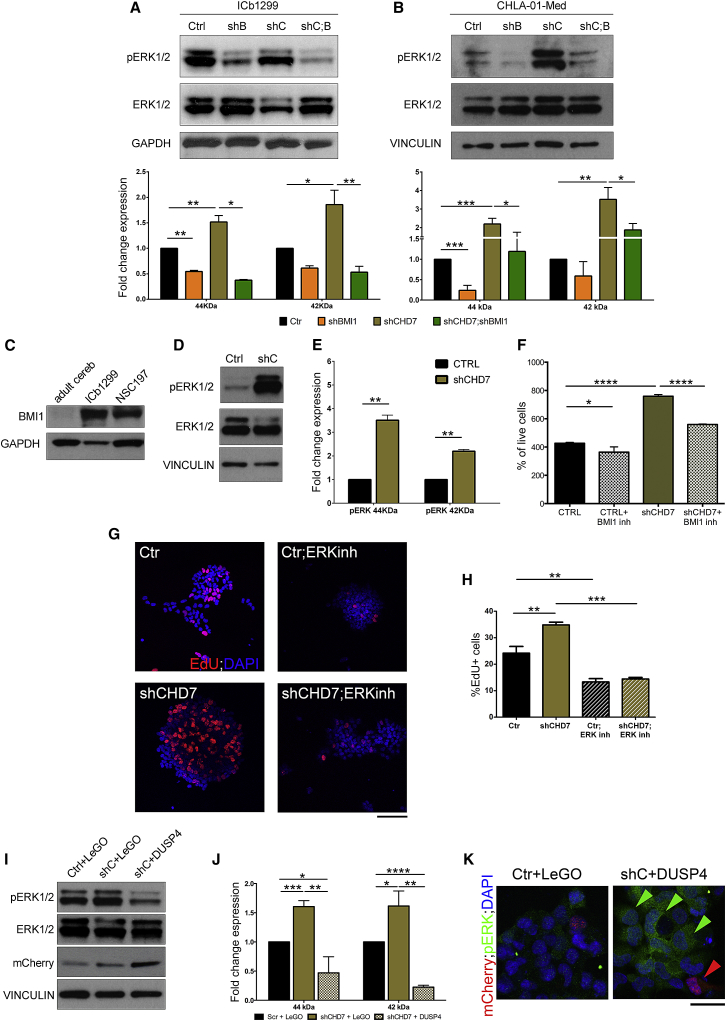
The Pro-proliferative State of the G4 MB Cells upon Silencing of CHD7 Is Mediated by ERK1/2 (A) Overactivity of the ERK1/2-signaling pathway in ICb1299 CHD7-silenced cells is neutralized by concomitant BMI1 silencing. (B) Similar effects are seen in CHLA-01-Med. (C) BMI1 expression in G4 MB cells and in NSC197 is higher than adult cerebellum, as shown by western blot analysis. (D and E) Silencing of CHD7 in NPCs leads to ERK1/2 pathway overactivation, as shown by western blot analysis (D) and quantification (E) (n = 2). (F) Increased proliferation is seen upon CHD7 silencing in NPCs, a finding that is neutralized by incubation with a BMI1 inhibitor (n = 3). (G) Increased proliferation as assessed by EdU pulse/chase is dependent on ERK1/2 overactivity, as demonstrated by its reduction upon pharmacological inhibition of ERK in ICb1299. (H) Quantification of the findings. (I and J) Overexpression of exogenous mCherry-DUSP4 abolishes ERK signaling overactivation mediated by CHD7 silencing. Western blot analysis (I) of ICb1299 transduced with empty LeGO control or LeGO-mCherry-DUSP4 and quantification (J) (n = 3). (K) Immunofluorescence of ICb1299 transduced as described in (I). Green arrows indicate CHD7-silenced cells with ERK overactivation, while red arrow indicates a cell overexpressing DUSP4 and devoid of ERK phosphorylation. Scale bar represents 25 μm. Data are represented as mean ± SEM. See also Figure S5.

Western blot analysis for pERK1/2 and ERK1/2 demonstrated pathway activation under conditions where BMI1 was expressed and in a more pronounced fashion when CHD7 was silenced (Figures 5A and 5B), in keeping with *DUSP4* gene repression by BMI1. Indeed, knockdown of BMI1 significantly reduced ERK1/2 pathway activation in G4 MB lines (Figures 5A and 5B), as predicted and in line with the upregulation of *DUSP4* observed in the genome-wide comparative expression analysis. Importantly, no overactivity of ERK signaling was found in the G3 MB line (Figure S5A), in keeping with no evidence of DUSP4 upregulation (Figure S4C). These data suggest a model where CHD7 silencing increases chromatin accessibility at the *DUSP4* locus to facilitate BMI1-mediated repression and consequent ERK pathway activation in G4 MB. Under conditions where both BMI1 and CHD7 are silenced, ERK pathway activation is down to baseline levels, a finding that is unlikely to be mediated by DUSP4, as BMI1 and CHD7 are silenced and the chromatin configuration at the locus is closed (Figure 4E).

To determine whether this BMI1-CHD7 connection also applied to freshly isolated cells and is not a feature of cells maintained through several *in vitro* passages, we obtained neural progenitor cells (NPCs) from human fetal brains. These cells expressed high levels of BMI1 as compared to adult cerebellum, albeit not as high as G4 MB cells (Figure 5C; Figure S5C). We found that the ERK1/2 pathway activity was increased (Figures 5D and 5E) upon CHD7 silencing (Figure S5D), which led to increased proliferation in a BMI1-dependent fashion also in these cells (Figure 5F).

Next, we assessed whether activation of ERK signaling was an essential mediator of the pro-proliferative phenotype observed upon silencing of CHD7 in G4 MB cells. Treatment of ICb1299 cells with an ERK inhibitor effectively inhibited the pathway (Figure S5E) and led to neutralization of the increased proliferation observed upon silencing of CHD7, an effect that was also observed in control cells (Figures 5G and 5H). Re-expression of CHD7 in ICb1299 rescued the pro-proliferative phenotype induced by CHD7 knockdown (Figures S3D–S3F), and it neutralized the ERK pathway overactivity in these cells (Figure S5B).

Finally lentiviral-mediated overexpression of mCherry-DUSP4 (Figure S5F) in ICb1299 abolished the ERK overactivation mediated by CHD7 silencing (Figures 5I–5K).

In conclusion, these data highlight a mechanism mediating a pro-proliferative role for BMI1/CHD7 in MB G4, which impacts ERK and is regulated by DUSP4.

### CHD7 Controls Tumor Volume in a BMI1/pERK-Dependent Fashion in Xenograft Models

**Figure 6:**
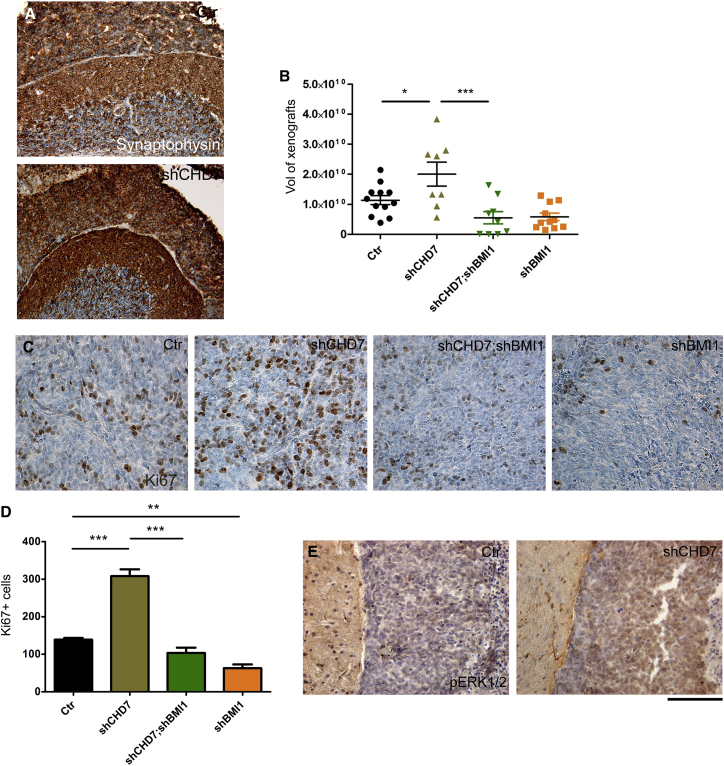
Increased Tumor Volume in CHD7-Silenced Xenografts of G4 MB Is BMI1 Dependent (A) Orthotopic xenografts of G4 MB cells silenced for CHD7 or CHD7;BMI1 or BMI1 show similar morphology, and they express Synaptophysin. (B) Increased tumor volume upon injection of shCHD7 G4 MB cells, which is neutralized by concomitant silencing of BMI1. (C) Increased proliferation, as assessed by Ki67 staining, in shCHD7 xenografts is shown together with its BMI1 dependency. (D) Quantification of the findings. (E) Overactivation of the ERK pathway is seen in shCHD7 xenografts. Scale bar represents 2 mm (A) and 125 μm (C and E). Data are represented as mean ± SEM. See also Figure S6.

To evaluate the *in vivo* relevance of these findings, we injected shCHD7 ICb1299 cells, as well as shCHD7;shBMI1 and shBMI1 cells, into the cerebellum of newborn mice. Xenografted mice were kept on tumor watch until symptoms of increased intracranial pressure developed, when they were culled. Histological analysis of the tumors did not reveal morphological differences between MBs originating from the different conditions, including the expression of markers such as Synaptophysin (Figure 6A) and *OTX2* (Figure S6A) and the lack of expression of *GAB1* (Figure S6B) and *GLI1* (Figure S6C). Stereological assessment of tumor volume revealed larger MBs in mice engrafted with shCHD7 cells, an effect that was lost upon concomitant silencing of BMI1 in the engrafted cells (Figure 6B). Increased proliferation in shCHD7 tumors was confirmed with immunohistochemical detection of Ki67 (Figures 6C and 6D). Silencing of CHD7 did not affect intraparenchymal invasion, while silencing of BMI1 alone significantly hampered tumor cell invasion (Figure S6D), in keeping with previous reports (Merve et al., 2014). Overactivity of the ERK pathway was confirmed in the shCHD7 xenografts (Figure 6E).

Together, these data support the conclusion that CHD7 depletion enhances proliferation of G4 MB cells via a BMI1/pERK mechanism.

## Discussion

The PcG gene *BMI1* is upregulated in a variety of cancers, where it correlates with clinical grade/stage and a poor prognosis. Importantly, it is a highly druggable molecule; hence, understanding its mechanism of action in tumorigenesis will be essential to guide the further development and clinical testing of BMI1 pharmacological inhibitors.

Here we show that genome-wide *in vivo* insertional mutagenesis (T2Onc2) driven by the Sleeping Beauty (SB11) transposase in glutamatergic progenitor cells engineered to overexpress Bmi1 results in MB formation. In a Bmi1-overexpressing background, we observe frequent *T2Onc2*-inactivating insertions in the chromatin remodeling factor *Chd7* (Bajpai et al., 2010, Whittaker et al., 2017), suggesting that Bmi1 overexpression and Chd7 loss of function cooperate to induce MB. Importantly, we show the relevance of this molecular convergence for human MB. In fact, high expression of BMI1 in combination with low expression of CHD7 was found to be associated with a poor prognosis in human MBs, particularly those of the G4 subgroup, and loss-of-function mutations of *CHD7* are significantly enriched for in this subgroup.

The Sleeping Beauty transposon mutagenesis is a powerful *in vivo* screening tool for cancer gene discovery. In the context of MB, this tool has been successfully used to identify candidate genes, particularly in the context of SHH MB as expected when using Math1 drivers; however, many of the candidate genes identified with this tool are known to be overexpressed or silenced in non-SHH models of the human disease (Dey et al., 2013, Genovesi et al., 2013, Mumert et al., 2012, Wu et al., 2012). We have studied the molecular convergence of BMI1/CHD7 in G4 MB because of the results of mutation analysis in human tumors indicating that genetic lesions leading to reduced CHD7 expression are found almost exclusively in G4 MB—and never in SHH MB—and because of the negative prognostic correlation of the BMI1^High^;CHD7^Low^ signature found exclusively in G4 MB. Thus, while we initially sought to model the effects and consequences of BMI1 overexpression in an SHH model of MB, this forward genetic screen uncovered a molecular axis whose signaling modulates the pathogenesis of a proportion of human G4 MBs instead. Interestingly, there is mounting evidence of a glutamatergic cell of origin for at least a proportion of G4 MBs, as recently suggested by a computational reconstruction of core regulatory circuitry that identified subgroup-specific transcription factors (Lin et al., 2016), hence highlighting a potential ontogeny link between the discovery tool we used and the molecular signature we have identified. Further studies in genetically engineered mouse models will confirm or dispute this hypothesis.

We have used primary G4 MB cells to dissect the contribution of CHD7 to tumorigenesis, both *in vitro* and *in vivo* in a xenograft model. We show deregulated expression of neural differentiation markers upon silencing of CHD7, an effect that is in keeping with a pro-neurogenic role of CHD7 as previously described (Feng et al., 2013, Jones et al., 2015). Genome-wide analysis of gene expression upon CHD7 silencing reveals many more upregulated genes as compared to downregulated genes, in keeping with CHD7 functioning as a repressor in our experimental setting. This is in agreement with a reported role of CHD7 as a repressive modulator of embryonic stem cell (ESC)-specific gene expression via targeting enhancer regions (Schnetz et al., 2010). In keeping with CHD7 functioning as a repressor in our experimental setup, no synergism between CHD7 and SOX2 was observed, an interaction known to lead to gene activation (Engelen et al., 2011). SOX4 and 11 are also unlikely to mediate CHD7 function in our setting, because CHD7 promotes an open chromatin configuration at their promoters (Feng et al., 2013).

We show increased proliferation of G4 MB cells upon CHD7 silencing *in vitro* as well as increased tumor volume upon orthotopic xenografting of these cells into NOD/SCID mice, an effect that is dependent on BMI1 expression. Bioinformatic analysis of the deregulated genes identified in a comparative gene expression analysis with human MB datasets revealed a BMI1-dependent convergence on modulation of ERK signaling via repression of DUSP4 in CHD7-depleted cells. Meta-analysis of publicly available ChIP-seq and RNA-seq datasets (Gargiulo et al., 2013) confirmed that DUSP family members are direct target genes of Bmi1. We provide evidence of altered chromatin accessibility at the promoter of *DUSP4* upon CHD7 silencing. This is in keeping with the recently reported ability of CHD7 to alter chromatin accessibility in primary cerebellar granule neuron progenitors (Feng et al., 2017, Whittaker et al., 2017), and it provides support to the notion that PcG-mediated gene repression is counteracted by a compacted chromatin configuration induced by CHD7 in G4 MB cells. Our findings are in good agreement with a previously reported role for DUSP4 in promoting neuronal differentiation and repressing proliferation via repression of the ERK pathway (Kim et al., 2015). Furthermore, activation of ERK signaling has been shown to control a proneural genetic program during cortical development and to activate an Ascl1-controlled neuronal differentiation program (Li et al., 2014). Overactivity of the pathway was found to exert a pro-proliferative role in SHH MBs (Brechbiel et al., 2014, Sengupta et al., 2012) and in other brain tumors, pilocytic astrocytomas, and glioneuronal tumors (Li et al., 2014). We show that activation of ERK signaling via the repression of DUSP4 by BMI1 is not found in a G3 MB line, lending additional support to the conclusion that this molecular convergence is functionally relevant only in a subset of G4 MBs. Pharmacological inhibition of ERK signaling confirmed the functional relevance of this regulatory loop in the G4 MB lines analyzed. Finally, silencing CHD7 in human neural progenitor cells expressing high levels of BMI1 induced overactivation of the ERK pathway and increased proliferation of these cells *in vitro*. These data raise the possibility that a BMI1^High^;CHD7^Low^ signature is oncogenic in human progenitor cells; genetically engineered models recapitulating this signature in a spatiotemporally controlled fashion will be essential to test this hypothesis.

The reversible nature of epigenetic modifications makes them attractive therapeutic targets, and pharmacological agents that can potentially reverse altered chromatin states are undergoing trials. Our findings extend the current knowledge of the role of two essential chromatin modifiers, BMI1 and CHD7, in MB pathogenesis, and they raise the possibility that pharmacological targeting of BMI1 may be particularly indicated in a subgroup of MB with low expression level of CHD7. Alternatively, the use of ERK inhibitors could also be further explored for MB treatment, and we provide here essential preclinical evidence that BMI1 and CHD7 could be useful biomarkers to define the patient subgroup that could benefit from this approach.

## Experimental Procedures

Detailed methods are available in the Supplemental Experimental Procedures.

### Sleeping Beauty Mutagenesis

Male and female Math1-Bmi1, Math1-Bmi1;SB11;T2Onc2, or T2Onc2 mice (12 to 20 weeks of age; at the time they developed signs of MB) were used. We did not perform a formal sample size estimate for the study but based our experimental plan on our previous experience with Sleeping Beauty mutagenesis screening. When mice showed clinical signs of increased intracranial pressure, they were culled, the brains were removed, and they were either fixed in formalin for histological assessment or used for genomic DNA extraction. All the procedures involving animals have been approved by the institutional Animal Care Committee and in no case were tumor-bearing animals allowed to reach a tumor burden compromising normal behavior, food and water intake, or exceeding the approved volume of 1,700 mm^3^.

### Culture Conditions for Patient-Derived MB Lines

Patient-derived MB lines were obtained from Dr Xiao-Nan Li, Baylor College of Medicine, Texas Children Cancer Centre, USA (Shu et al., 2008, Zhao et al., 2012). ICb1299 was cultured in DMEM (high glucose, GlutaMAX, Thermo Fisher Scientific) supplemented with 10% fetal bovine serum and 1% penicillin-streptomycin. ICb1595, ICb984, and ICb1338 were cultured in NeuroCult NS-A Basal Medium (human) (STEMCELL Technologies) supplemented with NeuroCult Proliferation Supplement (human) (STEMCELL Technologies), 1% penicillin-streptomycin, 2 μg/mL heparin (STEMCELL Technologies), 20 ng/mL epidermal growth factor (EGF) (recombinant mouse, PeproTech), and 10 ng/mL basic fibroblast growth factor (bFGF) (human recombinant, PeproTech). ICb1595, ICb984, and ICb1338 were cultured on plates coated with Poly-L-ornithine and Laminin (Sigma). CHLA-01-Med was purchased from ATCC (CRL3021) and grown in DMEM-F12 (Gibco) supplemented with B27 (Gibco), 20 ng/mL EGF, and 10 ng/mL bFGF. Cells were maintained at 37°C and were sub-cultured every 3 days once they reached confluence.

### Proliferation Assay

Patient-derived cells infected with lentiviral constructs coding for shBMI1 and/or shCHD7 were analyzed with cell growth curve and EdU staining to assess their proliferation potential compared to scramble control.

### *In Vivo* Orthotopic Xenografts

All procedures had Home Office approval (Animals Scientific Procedures Act 1986, PPL 70/7275) and were carried out as previously reported (Merve et al., 2014). Mice were killed when developing neurological signs and brains were removed and placed in 10% formalin for 24 hr and then transferred to PBS.

### Inhibition of MEK/ERK Activity

Patient-derived cells were treated with the MEK inhibitors PD98059 (Cell Signaling Technology) or U0126 (Cell Guidance Systems) at different concentrations. After 1 hr of treatment, cells were harvested and lysed for protein analysis. Alternatively, to assess proliferation potential, cells were treated twice with U0126 (50 μM) before pulsing with EdU or collecting them for western blot analysis.

### Western Blot Analysis

Total protein extracts were prepared with radioimmunoprecipitation assay (RIPA) buffer supplemented with protease inhibitors. Nuclear protein extracts were obtained as previously described (Badodi et al., 2015). Equal amounts of protein extracts were separated by SDS-PAGE and analyzed by western blot analysis.

### Statistical Analysis

Differences between groups were determined by one-way ANOVA, followed by Dunnett’s post hoc test (ATAC experiments) or Tukey’s multiple comparison test (all other experiments) to determine which groups differed significantly from control. Any p values < 0.05 were considered significant (^∗^p < 0.05, ^∗∗^p < 0.01, ^∗∗∗^p < 0.001, and ^∗∗∗∗^p < 0.0001).

## Author Contributions

S.M. and M.D.T. conceived the project. S.B., P.G., M.L., M.A.B., M.D.T., and S.M. designed the experiments. S.B., X.Z., A.D., A.M., P.S., M.D.C.J., M.M.K.-S., and M.A.B. conducted the wet lab experiments. S.B., X.Z., A.D., A.M., P.S., L.G., M.D.C.J., M.A.B., M.D.T., M.M.K.-S., S.K.S., and S.M. analyzed the data. X.-N.L. provided molecularly characterized primary MB lines. A.S.M., H.F., and G.R. conducted and analyzed the computational experiments. S.B. and S.M. wrote the paper with contributions from all authors.

## Supplemental Information

Document S1. Supplemental Experimental Procedures and Figures S1–S6

Data S1. Bmi1-SB versus SB and Ptc-SB Analysis: Comparative Insertion Site Analysis, Related to Figure 1

Data S2. Bmi1 filtered.clonal.gCIS_3: Insertion Sites in MB Originating in *Math1Bmi1;SB11;T2Onc2*, Related to Figure 1

Document S2. Article plus Supplemental Information
