# Supplementary material for: Convergence of BMI1 and CHD7 on ERK Signaling in Medulloblastoma
Source: Cell Rep. 2017 Dec 5;21(10):2772–84. doi: 10.1016/j.celrep.2017.11.021 (PMC5732319; doi:10.1016/j.celrep.2017.11.021)
Supplement: Document S1. Supplemental Experimental Procedures and Figures S1–S6 [file mmc1.pdf]

Cell Reports, Volume *21*

Supplemental Information

Convergence of BMI1 and CHD7 on ERK Signaling in Medulloblastoma

Sara Badodi, Adrian Dubuc, Xinyu Zhang, Gabriel Rosser, Mariane Da Cunha Jaeger, Michelle M. Kameda-Smith, Anca Sorana Morrissy, Paul Guilhamon, Philipp Suetterlin, Xiao-Nan Li, Loredana Guglielmi, Ashirwad Merve, Hamza Farooq, Mathieu Lupien, Sheila K. Singh, M. Albert Basson, Michael D. Taylor, and Silvia Marino

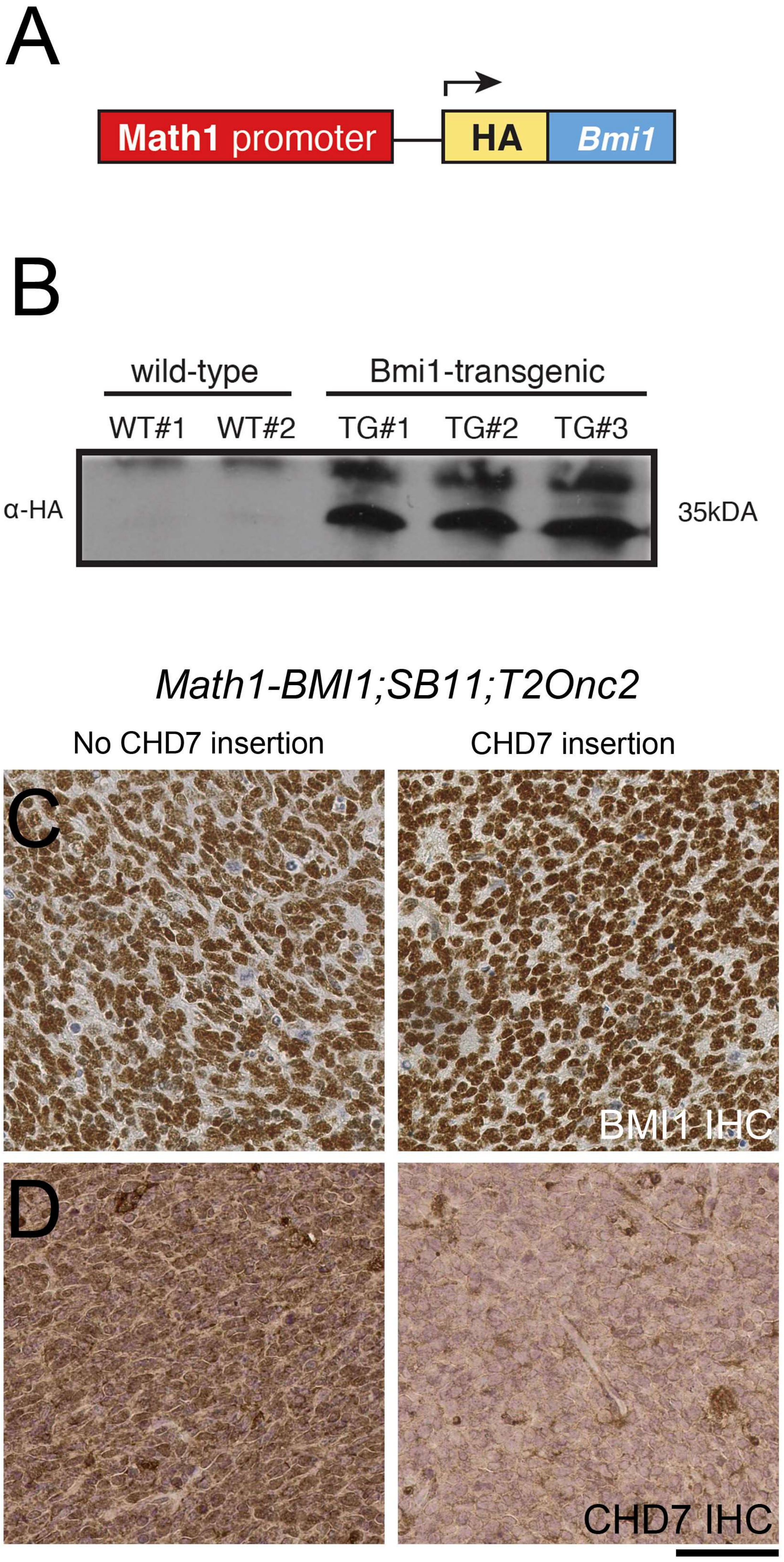
A
Math1 promoter
HA
Bmi1
B
wild-type
Bmi1-transgenic
WT#1
WT#2
TG#1
TG#2
TG#3
α-HA
35kDA
Math1-BMI1;SB11;T2Onc2
No CHD7 insertion
CHD7 insertion
C
BMI1 IHC
D
CHD7 IHC

Figure S1. Characterisation of M*ath1Bmi1 and MathBmi1;SB11;T2Onc2* mice. Related to Figure 1. A: Construct of genomic sequence used to generate Bmi1 transgenic/overexpressing animal model under the MATH1-promoter/enhancer. **B:** Anti-HA western blot validating the overexpression of Bmi1 in transgenic (TG) lines as compared to wild-type (WT) littermates. **C:** Expression of Bmi1 in SB MB. **D:** Low expression of Chd7 in SB MB with inactivating insertion in the Chd7 locus. Scale bar is 125 μm.

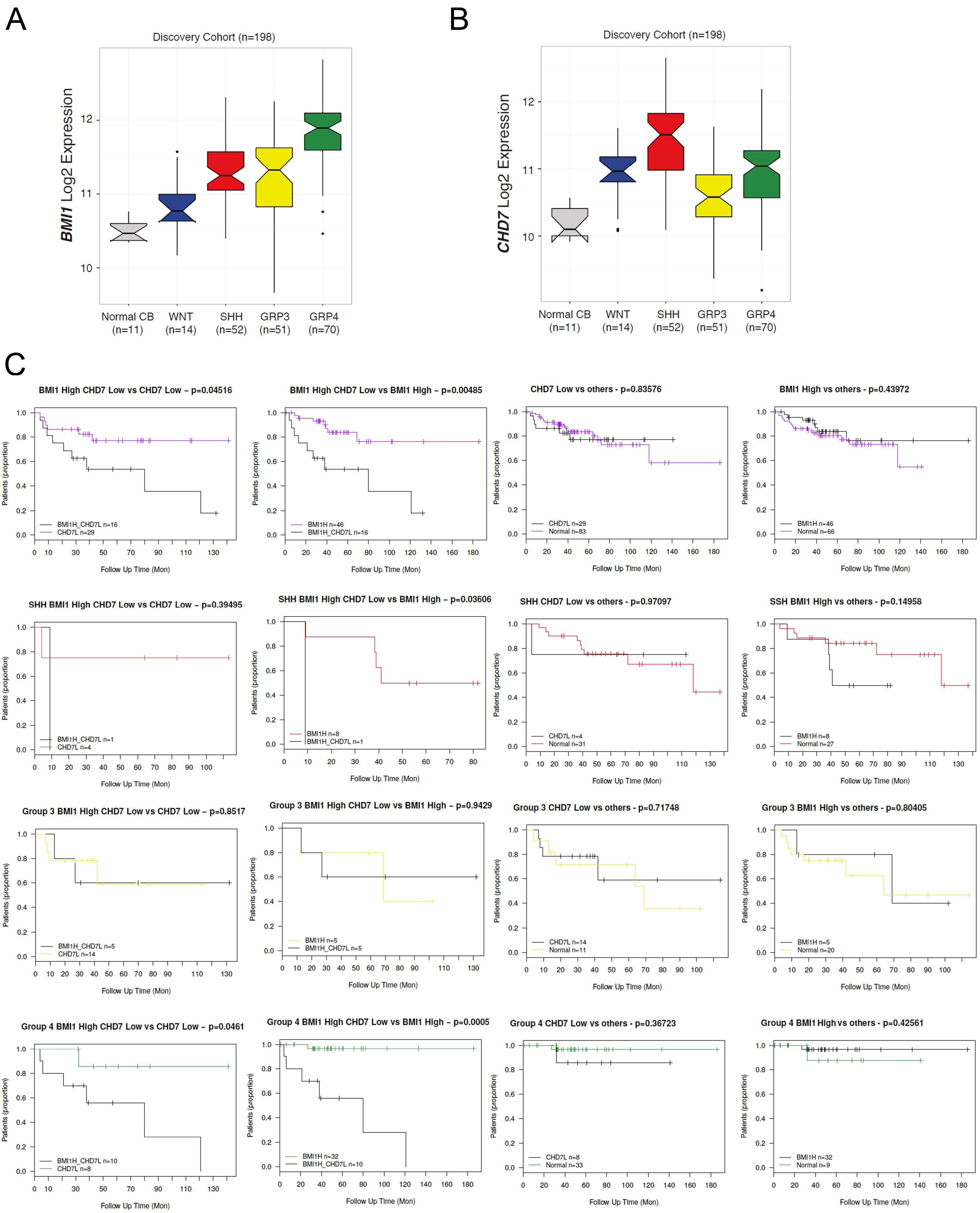

A
Discovery Cohort (n=198)
BMI1 Log2 Expression
Normal CB (n=11)
WNT (n=14)
SHH (n=52)
GRP3 (n=51)
GRP4 (n=70)
B
Discovery Cohort (n=198)
CHD7 Log2 Expression
Normal CB (n=11)
WNT (n=14)
SHH (n=52)
GRP3 (n=51)
GRP4 (n=70)
C
BMI1 High CHD7 Low vs CHD7 Low – p=0.04516
BMI1H_CHD7L n=16
CHD7L n=29
BMI1 High CHD7 Low vs BMI1 High – p=0.00485
BMI1H n=46
BMI1H_CHD7L n=16
CHD7 Low vs others - p=0.83576
CHD7L n=29
Normal n=83
BMI1 High vs others - p=0.43972
BMI1H n=46
Normal n=66
SHH BMI1 High CHD7 Low vs CHD7 Low – p=0.39495
BMI1H_CHD7L n=1
CHD7L n=4
SHH BMI1 High CHD7 Low vs BMI1 High – p=0.03606
BMI1H n=8
BMI1H_CHD7L n=1
SHH CHD7 Low vs others - p=0.97097
CHD7L n=4
Normal n=31
SSH BMI1 High vs others - p=0.14958
BMI1H n=8
Normal n=27
Group 3 BMI1 High CHD7 Low vs CHD7 Low – p=0.8517
BMI1H_CHD7L n=5
CHD7L n=14
Group 3 BMI1 High CHD7 Low vs BMI1 High – p=0.9429
BMI1H n=5
BMI1H_CHD7L n=5
Group 3 CHD7 Low vs others - p=0.71748
CHD7L n=14
Normal n=11
Group 3 BMI1 High vs others - p=0.80405
BMI1H n=5
Normal n=20
Group 4 BMI1 High CHD7 Low vs CHD7 Low – p=0.0461
BMI1H_CHD7L n=10
CHD7L n=8
Group 4 BMI1 High CHD7 Low vs BMI1 High – p=0.0005
BMI1H n=32
BMI1H_CHD7L n=10
Group 4 CHD7 Low vs others - p=0.36723
CHD7L n=8
Normal n=33
Group 4 BMI1 High vs others - p=0.42561
BMI1H n=32
Normal n=9
Patients (proportion)
Follow Up Time (Mon)

Figure S2. Characterisation of BMI1 and CHD7 expression levels across MB subgroups. Related to Figure 2. A-B: Box plot representation of BMI1 expression across a cohort of 198 primary MB profiled on Affymetrix 1.1ST gene arrays. BMI1 was significantly overexpressed in G4 MBs versus other molecular subgroups. Conversely, CHD7 was significantly downregulated in G4 versus other molecular subgroups. **C:** Kaplan Meier survival analysis of patients with the BMI^{High};$CHD7^{Low}$ signature relative to BMI^{High} only and $CHD7^{Low}$ only. Across the full cohort, the BMI^{High};$CHD7^{Low}$ signature is associated with significantly poorer outlook compared to the single phenotypes. Dividing the cohort into subgroups demonstrates that this finding is consistently reproduced only in G4.

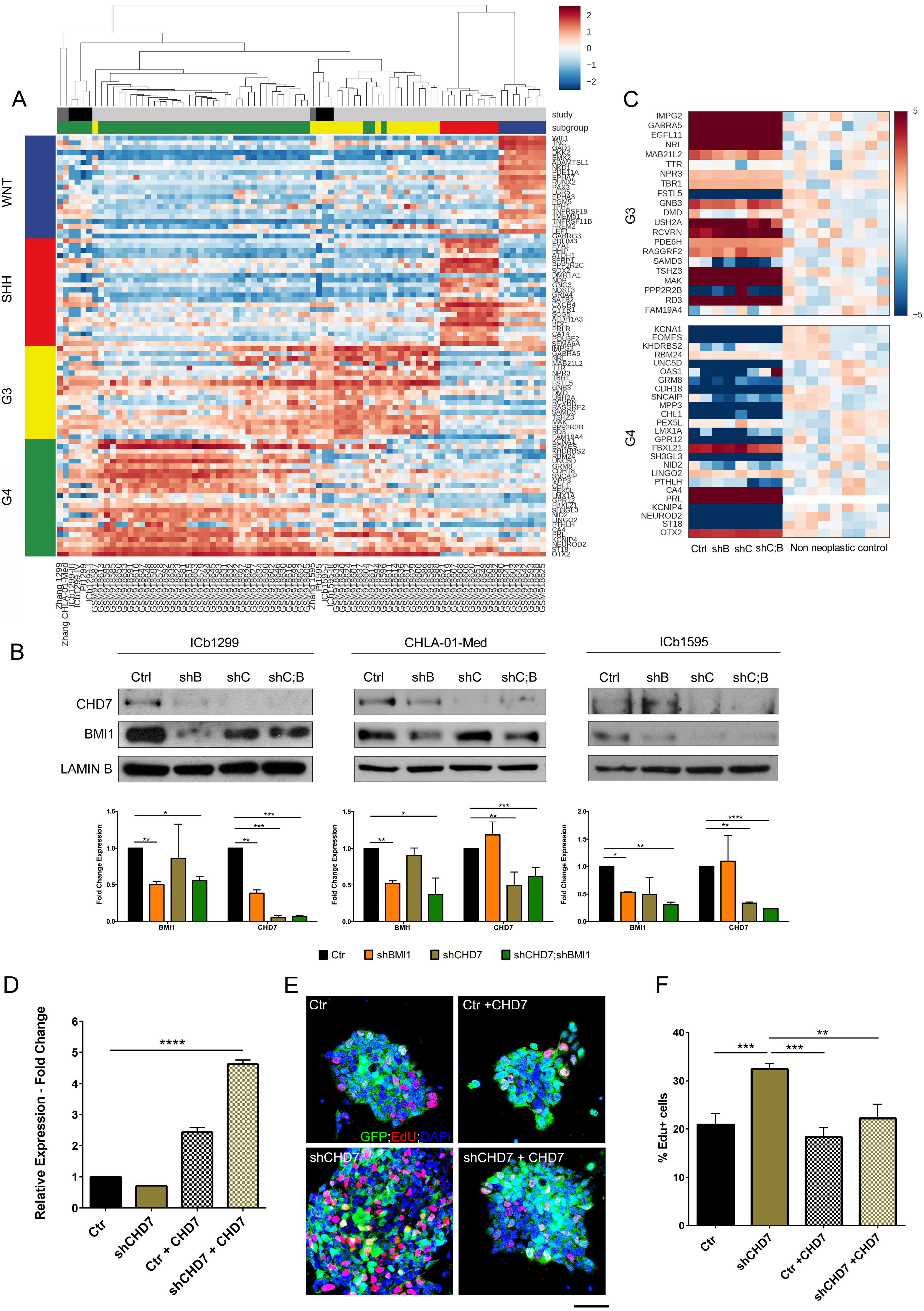
A
B
C
D
E
F
study
subgroup
WNT
SHH
G3
G4
ICb1299
CHLA-01-Med
ICb1595
Ctrl
shB
shC
shC;B
CHD7
BMI1
LAMIN B
Fold Change Expression
Ctr
shBMI1
shCHD7
shCHD7;shBMI1
Non neoplastic control
Relative Expression - Fold Change
Ctr + CHD7
shCHD7 + CHD7
Ctr +CHD7
GFP;EdU;DAPI
% Edu+ cells
shCHD7 +CHD7

Figure S3. Patient-derived MB cells retain their subgroup affiliation and reconstitution of CHD7 expression in shCHD7. Related to Figure 3. A: Heatmap showing relative gene expression for 100 medulloblastoma signature genes (Lin et al., 2016) for the Robinson cohort (light grey), tumour and early passage xenograft culture data from Zhao et al. (black; tumour samples have names beginning Pt) and the current study (dark grey). The dashed line indicates that 5 clusters are detected. The Robinson data are clustered into the expected subgroups with three errors. ICb1595 cluster with other G3 samples. ICb1299 and CHLA-01-Med cluster separately but closer to G3 and G4 than WNT and SHH. **B:** Expression levels of BMI1 and CHD7 upon silencing of BMI1 or CHD7 or both (n = 3). **C:** Log2 gene expression levels for 50 signature genes commonly associated with G3 and G4 medulloblastoma in the single, double and control knockdown conditions of line ICb1299 (in duplicate), relative to healthy cerebellum tissue. The ICb1299 samples show a consistent profile across all conditions. D: Reconstitution of CHD7 expression is shown by qRT-PCR in ICb1299 G4 MB where CHD7 had been previously silenced. **E:** Increased proliferation as assessed by EdU pulse/chase upon CHD7 silencing is rescued upon reconstitution of CHD7 expression in ICb1299. **F:** Quantification of the findings. Data are represented as mean ± SEM. Scale bar is 50 μm.

A

gene	shCHD7	shBMIshCHD7	Icb1299_FC	Icb1299_p_value	Icb1299_q_value	MB_FC_ratio	MB_p_value	MB_sig	MB_sig	Status
SLC35D1	3.00135	1.96715	-1.525735201	0.04215	0.125181	-1.078730405	0	yes	yes	DOWN
DGKI	3.57766	1.08168	-3.307503143	0.0237	0.10338	-1.042759699	0.004	yes	yes	DOWN
SLC24A3	5.85064	3.49868	-1.672242103	0.0182	0.0994977	-1.130926405	0.004	yes	yes	DOWN
ID2	15.3534	10.0514	-1.527488708	0.0451	0.13191	-1.128258979	0.004	yes	yes	DOWN
GALNT1	19.5259	11.5071	-1.696856723	0.0023	0.0437563	-1.035042798	0.007	yes	yes	DOWN
CNTN1	39.1922	27.1748	-1.442225886	0.01705	0.0947374	-1.248564472	0.007	yes	yes	DOWN
PARM1	11.489	7.49711	-1.532457173	0.01285	0.0833449	-1.116507523	0.011	yes	yes	DOWN
HIF1A	13.0248	7.27331	-1.790766515	0.0048	0.0585563	-1.018513181	0.026	yes	yes	DOWN
SP4	2.95444	1.81172	-1.630737642	0.02205	0.100642	-1.052829404	0.028	yes	yes	DOWN
PPT1	46.3811	31.2354	-1.48488894	0.0115	0.0769811	-1.03633594	0.033	yes	yes	DOWN
PPP2R2B	33.5442	21.4357	-1.564875418	0.0143	0.0851283	-1.11565787	0.04	yes	yes	DOWN
SEMA3A	2.4877	1.03614	-2.400930376	0.00065	0.0342897	-1.230436811	0.041	yes	yes	DOWN
PRNP	85.2033	59.8327	-1.424025658	0.021	0.100642	-1.029101168	0.042	yes	yes	DOWN
COL8A2	1.897	1.11297	-1.704448458	0.047	0.136725	-1.038633848	0.045	yes	yes	DOWN
CGGBP1	29.3201	19.3095	-1.518428753	0.01535	0.0876301	-1.072158891	0.049	yes	yes	DOWN
TMEM158	17.167	25.0201	1.457453253	0.0384	0.122867	1.168853411	0	yes	yes	UP
TIMM10	76.3064	129.571	1.698035814	0.0022	0.0437563	1.048232718	0.021	yes	yes	UP
UCP2	19.7778	29.1342	1.473075873	0.03025	0.115224	1.071493335	0.037	yes	yes	UP
DUSP4	5.55553	8.17981	1.472372573	0.0265	0.108734	1.141653888	0.041	yes	yes	UP
PTTG1	126.979	191.479	1.507958009	0.01025	0.0761169	1.070075107	0.048	yes	yes	UP

B

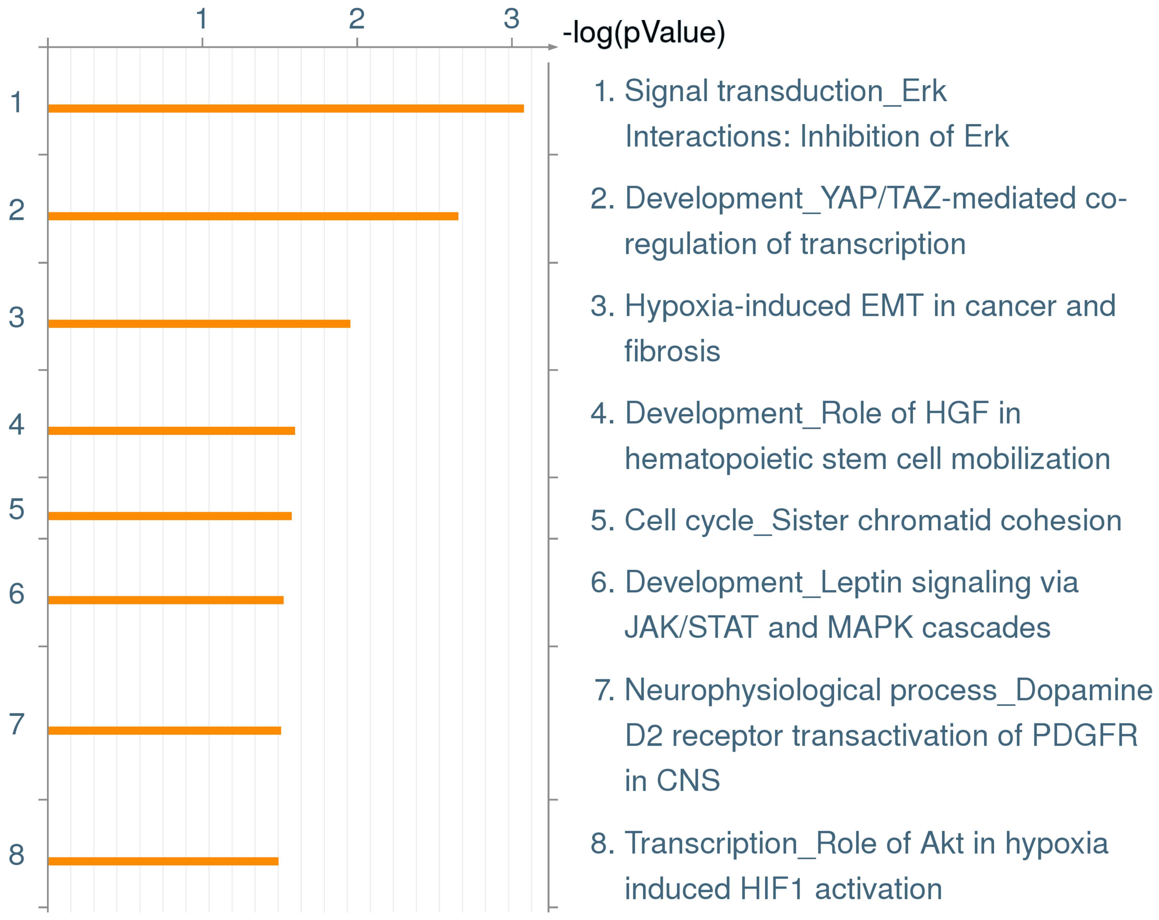

C

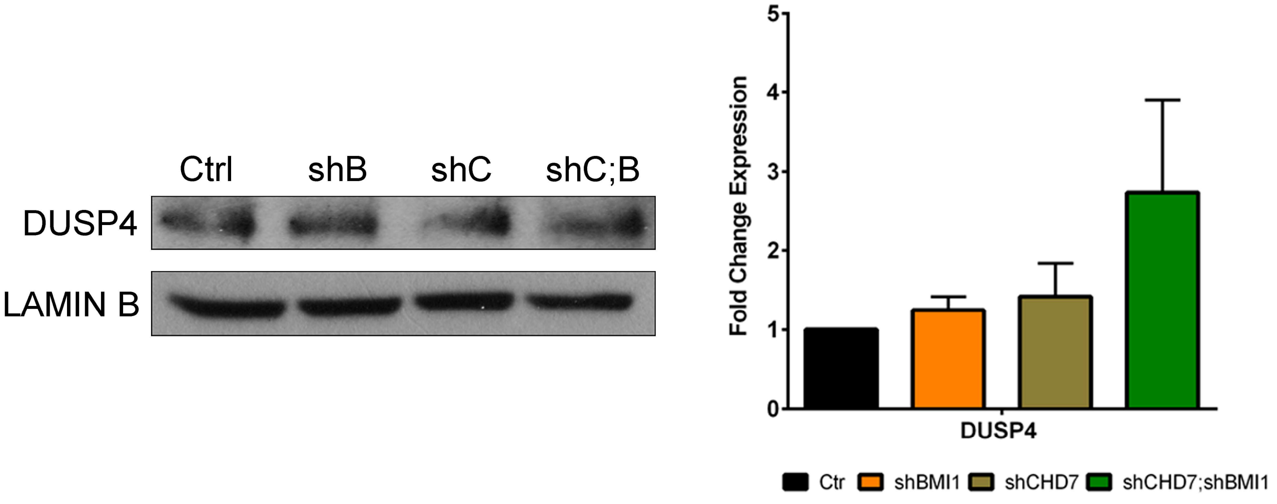

Figure S4. Identification of ERK signal transduction as specifically affected in BMI1^{High};CHD7^{Low} MB G4 and primary lines. Related to Figure 4. **A:** Summary table of DE genes in G4 MB cells upon BMI1 knockdown in a CHD7 silenced context and in G4 MB with a BMI^{High};CHD7^{Low} signature. **B:** Metacore analysis of the 20 DE genes shows an impact on ERK signalling. **C:** Western blot and quantification of DUSP4 expression show that BMI1 doesn't regulate DUSP4 in G3 MB cells (n = 3). Data are represented as mean ± SEM.

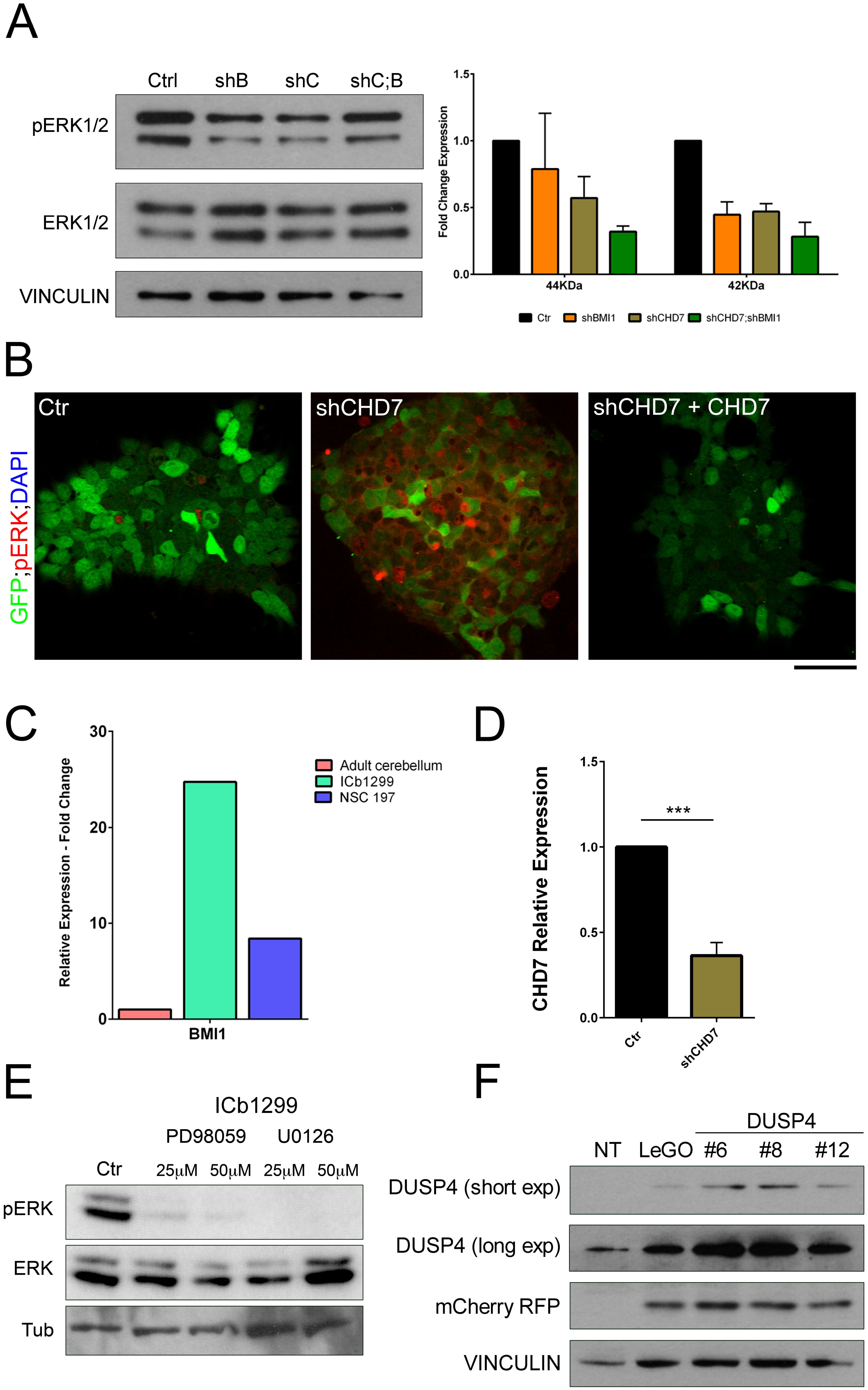
A
Ctrl
shB
shC
shC;B
pERK1/2
ERK1/2
VINCULIN
Fold Change Expression
1.5
1.0
0.5
0.0
44KDa
42KDa
Ctr
shBMI1
shCHD7
shCHD7;shBMI1
B
Ctr
shCHD7
shCHD7 + CHD7
GFP;pERK;DAPI
C
Relative Expression - Fold Change
30
20
10
0
BMI1
Adult cerebellum
ICb1299
NSC 197
D
CHD7 Relative Expression
1.5
1.0
0.5
0.0
***
Ctr
shCHD7
E
ICb1299
PD98059
U0126
Ctr
25µM
50µM
25µM
50µM
pERK
ERK
Tub
F
DUSP4
NT
LeGO
#6
#8
#12
DUSP4 (short exp)
DUSP4 (long exp)
mCherry RFP
VINCULIN

Figure S5. The pro-proliferative state mediated by shCHD7 is specific for MB G4 and is mediated by ERK1/2 via DUSP4 regulation. Related to Figure 5. **A**: No overactiviation upon CHD7 silencing in G3 MB cells. (n=2). **B:** Immunofluorescence analysis showing rescue of the ERK pathway overactivation upon CHD7 reconstitution in ICb1299. Scale bar is 50 μm. **C:** Quantification of Western blot analysis in Fig. 5C shows overexpression of BMI1 in human NPC as compared to adult cerebellum. **D:** Efficient silencing of CHD7 in the cells analysed in Fig. 5D and E. **E:** Western Blot analysis showing effective pharmacological inhibition of ERK1/2 pathway in ICb1299. **F:** Western blot analysis of HEK293 cells overespressing 3 different LeGO-mCherry-DUSP4 clones (#6, 8 and 12) shows increased levels of both mCherry and DUSP4 expression. Data are represented as mean ± SEM.

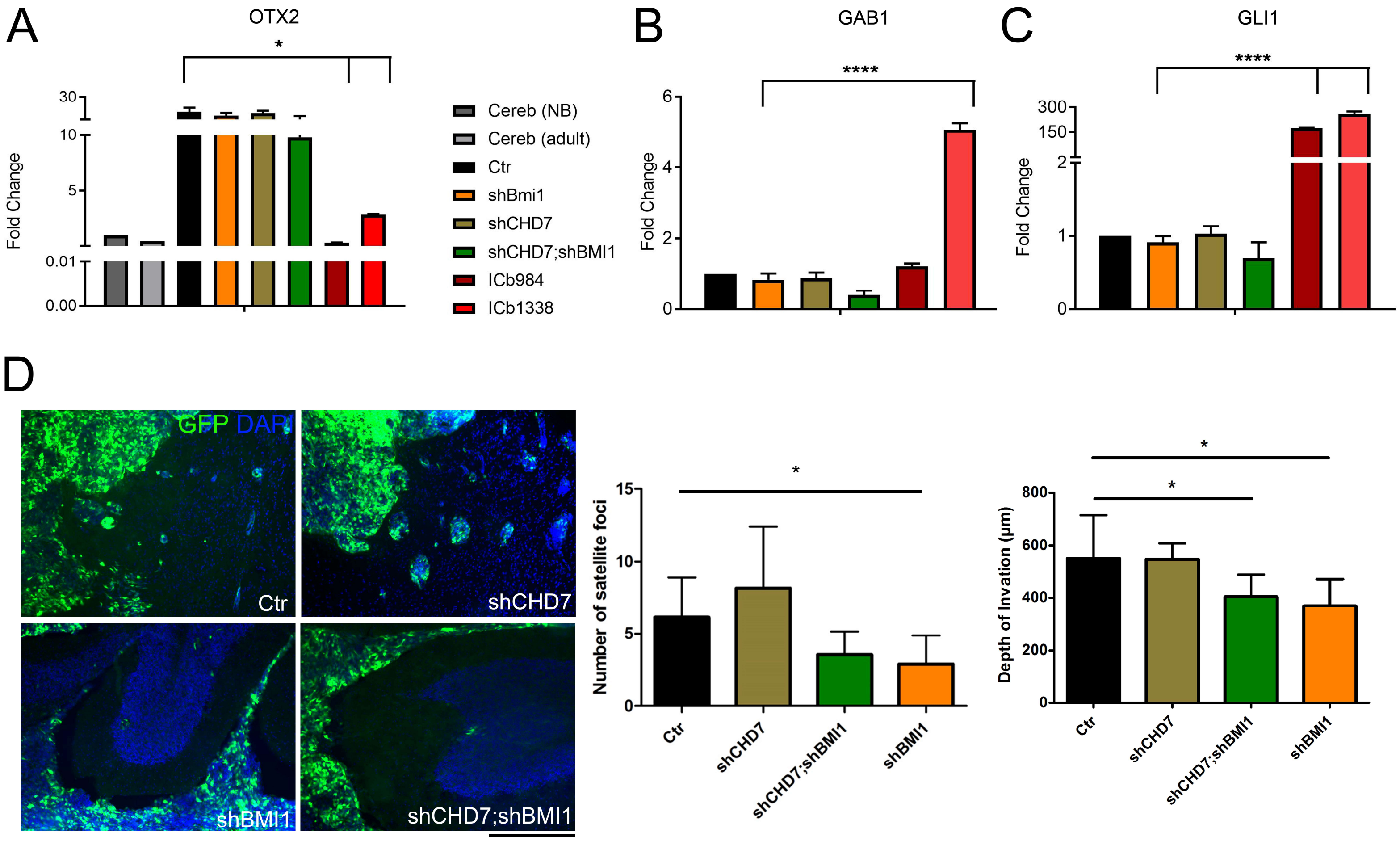

A
OTX2
*
30
10
5
0.01
0.00
Fold Change
Cereb (NB)
Cereb (adult)
Ctr
shBmi1
shCHD7
shCHD7;shBMI1
ICb984
ICb1338
B
GAB1
****
6
4
2
0
Fold Change
C
GLI1
****
300
150
2
1
0
Fold Change
D
GFP DAPI
Ctr
shCHD7
shBMI1
shCHD7;shBMI1
*
15
10
5
0
Number of satellite foci
Ctr
shCHD7
shCHD7;shBMI1
shBMI1
*
*
800
600
400
200
0
Depth of Invation (μm)
Ctr
shCHD7
shCHD7;shBMI1
shBMI1

Figure S6. Silencing of CHD7 did not affect intraparenchymal invasion and expression of subgroup specific genes in xenografts. Related to Figure 6. **A**: OTX2 expression is unchanged upon CHD7 and/or BMI1 silencing in G4 MB xenografts and is higher than normal cerebellum and SHH MB cells (ICb984 and ICb1338).

B-C: GAB1 and GLI1 expression in G4 MB xenografts is lower than SHH MB cells. **D:** Intraparenchymal invasion of G4 MB cells, as assessed by quantification of the number of satellite foci around the main tumour bulk (left) or as depth of invasion (right) is dependent on BMI1 but not on CHD7. Data are represented as mean ± SEM. Scale bar is 125 μm.

Extended Experimental procedures

Analysis of gCIS

A description of the linker-mediated PCR and Illumina HiSeq sequencing used for transposon insertion sites as well as details of the gCIS prediction, of the genetic algorithm for driver gene prediction and of the PCR for Sleeping Beauty tagged fragments are presented in (Morrissy et al., 2016). Briefly, sequenced libraries were demultiplexed and aligned as described (Brett et al., 2011). Custom scripts were used to demultiplex and trim SB transposon sequences, and reads were aligned to mouse assembly NCBI37/mm9 (July 2007) using Novoalign. Statistical enrichment of transposon integration events within each of 19,000 mouse RefSeq genes was assessed using a chi-squared test considering the following: the number of TA dinucleotide sites within the gene relative to the number of TA sites in the genome, the number of integration sites within each tumour, and the total number of tumours in each cohort. The p-value calculated for each of gene was adjusted for multiple hypothesis testing using Bonferroni correction. Finally, significant gCIS predictions were manually curated to filter out artefacts and local hopping events. The excel files of this analysis and of the comparative analysis with a published dataset of SB-induced insertion sites in MB occurring in Ptch1+/- mice (Morrissy et al., 2016) are included as supplementary material (Bmi1 filtered.clonal gCIS_3 and Bmi1-SB versus SB and Ptc-SB analysis-August 13-12).

DUSP4 lentiviral construct cloning

DUSP4 coding sequence was PCR cloned from plasmid R777-E039 Hs.DUSP4 (Addgene, 70323) and inserted in the lentiviral vector LeGO-iC carrying the fluorescent reporter RFP-mCherry with Gibson Assembly cloning strategy (NEB). The resulting *LeGO-iC-DUSP4-mCherry* was sequence verified.

Production of shRNA lentiviral vector and gene silencing

GIPZ lentiviral shRNA vectors containing a hairpin sequence targeting BMI1 or CHD7 and the coding sequences for GFP and puromycin resistence gene were purchased from Dharmacon, UK. Packaging, virus production and determination of titre were carried out as previously reported (Merve et al., 2014). Cells were infected overnight at a multiplicity of infection (MOI) of 12. After 96h from the infection, puromycin selection at a concentration of 2.5µg/ml was applied to enrich for the transduced population. The efficacy of gene silencing was assessed by qRT-PCR or Western blot analysis. All experiments were conducted at least in triplicates.

Growth Curve

Transduced patient-derived cells (Scramble control, shBMI1, shCHD7, shCHD7;shBMI1) were seeded in 24-well plates at the same density. For the following 10 days, cells were harvested at specific time points (1, 2, 3, 6 and 10 days) and the number of proliferating cells was counted with a hemocytometer and Trypan Blue staining. Experiments were carried out at least two times in triplicate.

EdU staining

The Click-iT® EdU Alexa Fluor® 594 Imaging Kit (ThermoFisher) was used to assess EdU incorporation following manufacturer recommendations. Briefly, transduced cells were seeded on coated coverslips in triplicates, treated with 10µM of EdU solution for 3 hours and then fixed in 4% paraformaldehyde (PFA). Cells were then washed, blocked and incubated with Click-iT® reaction cocktails containing Alexa Fluor® azide. Rabbit polyclonal GFP antibody (1:1000, ab290, abcam) was used to visualize transduced cells. Five representative images (40X magnification) of each sample were captured using a Zeiss 710 Confocal Microscope. The percentage of positive nuclei was calculated as the ratio between EdU-positive cells and the total number of nuclei counted using ImageJ software.

Human neural stem cells (NSCs) culture

Human fetal brains were obtained from consenting patients, as approved by Hamilton Health Sciences/McMaster Research Ethics Board. Samples were dissociated in PBS containing 0.2 Wunisch unit/mL Liberase Blendzyme (Roche), and incubated at 37^oC in a shaker for 15 min. The dissociated tissue was then filtered through 70µm cell strainer and collected by centrifugation at 1500 rpm for 3 min. Neural stem cells were resuspended in Neurocult complete media, (STEMCELL Technologies), supplemented with human recombinant epidermal growth factor (20ng/mL: STEMCELL Technologies), basic fibroblast growth factor (20ng/mL; STEMCELL Technologies), antibiotic-antimycotic (10mg/mL; Wisent), and plated in ultra-low attachment plates (Corning). Red cells were lysed using ammonium chloride solution (STEMCELL Technologies). Neural stem cells were grown in suspension in Neurocult complete for 2 days. Thereafter, the neurospheres were plated onto polyornithine-laminin (PL) coated tissue culture treated plates. Once propagated, the cells were dissociated with TrypLE (Invitrogen) and replated onto appropriately sized plates for subsequent experimentation.

NSCs transduction and propagation

NSCs were plated onto 24 well PL plates at a density of $2.0x10^5$ cells per well for transduction with appropriate viruses. The cells were incubated in 37^oC for 24 hours and the media was changed completely. Thereafter, the cells were incubated further in 37^oC for 48 hours. The transduced cells were selected with the addition of 1ug/mL puromycin in Neurocult complete media. The remaining cells after puromycin selection, were then propagated further and replated if necessary. Transduced NSC were dissociated and adjusted to 1 million single cells/mL in PBS+2mM EDTA. Samples were analysed and/or sorted using a MoFlo XDP cell sorter and Kaluza software (Beckman Coulter). Dead cells excluded using the viability dye 7-

AAD (1:10; Beckman Coulter). Cells were sorted into Neurocult complete media and allowed to equilibrate at 37°C overnight prior to use in experiments and replating onto PL plates.

RNA extraction and qRT-PCR analysis

Total RNA was isolated from cell pellets with RNeasy Micro purification kit (Qiagen) and digested with DNaseI (Applied Biosystems). 8 to 12 xenograft frozen sections were used to extract total RNA by the proteinase K/acid phenol method (Khodosevich et al., 2007) followed by digestion with DNaseI. Total RNA from formalin-fixed, paraffin-embedded (FFPE) samples was isolated with the FFPE RNA/DNA Purification Plus Kit (Norgen Biotek). 4 sections of 20 μm of thickness were used for each sample and DNaseI digestion was performed according to manufacturer instructions. The cDNA synthesis was carried out with SuperScript III Reverse Transcriptase Kit (Invitrogen). Analysis of gene expression was performed with the Applied Biosystems 7500 Real-Time PCR System using TaqMan gene expression MasterMix (Applied Biosystems) and SYBR Green PCR Master Mix (Applied Biosystems) according to standard protocols. Technical triplicates for each samples were analyzed. The Ct values of all the genes analyzed were normalized to the Ct of housekeeping gene (GAPDH for TaqMan probes and average Ct of 18S, ACTB and ATP 5B for SYBR Green PCR) and fold changes were calculated.

Target genes and TaqMan probes used are listed as below:

**Target Gene**	**Assay ID**
Bmi1	Hs00180411_m1
CHD7	Hs00215010_m1
EOMEOS	Hs00172872_m1

GAPDH	Hs02758991_g1
GAB1	Hs00157646_m1
GLI1	Hs00171790_m1
LHX2	Hs00180351_m1
LMX1A	Hs00892663_m1
ATOH1 (MATH1)	Hs00245453_s1
OTX2	Hs00222238_m1

Primers used in SYBR Green qPCR are the following:

BMI1 FW: GCTGGTTGCCCATTGACAG; REV: CGATGCATTTCTGCTTGATAA

CHD7 FW: GAAGAAGATATAGAGACCCCAC; REV: TCTTTGGTACATAACTTGGC

CHD7_NSC FW: CTTTTCATGAGCCACAAACG; REV: TCTTCTTCAAAAGCTTTGGTCAC

18S FW: CGCCGCTAGAGGTGAAATTCT; REV: CGAACCTCCGACTTTCGTTCT

ACT B FW: GCGAGAAGATGACCCAGATC; REV: CCAGTGGTACGGCCAGAGG

ATP 5B FW: CCCAGGCTGGTTCAGAGGT; REV: AGGGGCAGGGTCAGTCAAG

GAPDH_NSC FW: TGCACCACCAACTGCTTAGC; REV: GGCATGGACTGTGGTCATGAG

Immunocytochemistry (IHC)

IHC was conducted on FFPE or frozen sections of xenografts tumors (tissue fixed with 4% PFA and embedded in OCT) either manually or with an automated Ventana Discovery XT.

Manual IHC: Briefly, FFPE sections were dewaxed with xylene and hydrated with graded alcohol series. Heat-induced antigen unmasking was performed with Antigen Unmasking

Solution, citrate-based (Vector) and endogenous peroxidase activity was quenched with 3% H_2O_2 solution. Sections were treated with 10% Normal Donkey Serum to block non-specific binding sites and incubated overnight with primary antibodies at 4°C. Sections were then incubated with biotinylated secondary antibody (Vector, 1:500) for two hours at room temperature, treated with VECTASTAIN ABC Reagent (Vector) and DAB (SIGMAFAST tablets, Sigma Aldrich). Samples were counterstained with hematoxylin.

The following primary antibodies were used: rabbit polyclonal anti-CHD7 (1:500, ab117522, Abcam), rabbit monoclonal anti-phospho-p44/42 MAPK (1:100, D13.14.4E, Cell Signaling).

Automated IHC: Paraffin embedding, coronal sectioning of 3-µm and staining for haematoxylin and eosin, synaptophysin and human vimentin were performed by UCL IQPath (Institute of Neurology, London, UK). Immunostaining was done on Ventana Discovery XT instrument an automated staining machines (ROCHE, Burgess Hill, UK) following the manufacturer's guidelines, using horseradish-peroxidase-conjugated streptavidin complex and diaminobenzidine as a chromogen. The following antibodies were used for histological characterisation: synaptophysin (Invitrogen 080130, prediluted), KI67 and human vimentin (Roche 790-2917, prediluted).

Immunofluorescence analyses

Immunofluorescence analyses were conducted on Icb1299 cells and frozen tissue sections from xenograft tumours. Icb1299 cells, cultured on Poly-lysine (PLL) coated coverslips, were fixed using 4% PFA. Cells and freshly frozen tissue sections (xenografts fixed with 4% PFA and embedded in OCT) were treated with 10% Normal Goat Serum, followed by incubation with primary antibodies overnight at 4°C. Appropriate secondary antibodies (1:500, Invitrogen) were used. The samples were counterstained with DAPI. The following primary

antibody were used: mouse monoclonal anti-mCherry (1:200, ab125096, abcam); rabbit monoclonal anti-phospho-p44/42 MAPK (1:100, D13.14.4E, Cell Signaling); chicken polyclonal anti-GFP (1:500, ab13970, abcam).

Western blot analysis

Transduced patient-derived cells were lysed for 30 minutes on ice using RIPA lysis buffer supplemented with 2mM PMSF, 1mM sodium orthovanadate and protease inhibitor cocktail (PIC) (RIPA Lysis Buffer System, Santa Cruz) followed by 3 pulses of sonication. Frozen tissue was extensively washed with cold PBS supplemented with PIC to dissolve OCT before lysis in RIPA buffer.

Nuclear and cytoplasmic fractions of transduced cells were obtained with two different lysis buffers. Briefly cells were harvested in Buffer A (10 mM HEPES pH 7.9, 10 mM KCl, 0.1 mM EDTA, 0.15% Nonidet P40(NP40) and 0.1 mM EGTA) supplemented with 1 mM DTT and PIC. Cells were homogenized through a 26G needle, nuclei were isolated by centrifugation and the supernatant (cytoplasmic fraction) was collected. Nuclei were washed quickly with ice-cold PBS, suspended in Buffer B (20 mM HEPES pH 7.9, 400 mM NaCl, 1 mM EDTA, 1 mM EGTA and 0,5% NP 40) supplemented with 1 mM DTT and PIC and lysed by sonication. Protein concentration was determined using BCA Protein Assay Kit (Pierce). Equal amounts of protein were separated by SDS-PAGE and transferred onto nitrocellulose membrane (Amersham). After transfer, the membrane was blocked for one hour at room temperature in 5% skimmed milk in TBST buffer (25 mMTrisHCl, 137 mMNaCl, 0.1% Tween 20, pH 7.5) and probed with different antibodies. Incubation with primary antibody was performed overnight at 4°C followed by appropriate secondary HRP- conjugated antibodies (anti-rabbit IgG or anti-mouse IgG, 1:5000, Amersham) for one hour at room temperature. Enhanced chemoluminescence (ECL Plus, Amersham) was used for detection of the bands. The

following primary antibodies were used: mouse monoclonal anti-BMI1 (1:1000, clone AF27, Active Motif), anti-GAPDH (1:1000, G8795, Sigma), anti-mCherry (1:1000, ab125096, abcam), anti α-tubulin (1:5000, clone DM1A, Sigma) and anti-Vinculin (1:5000, V4505, Sigma); rabbit monoclonal anti-p44/42 MAPK (1:1000, 137F5, Cell Signaling) and anti-phospho-p44/42 MAPK (1:1000, D13.14.4E, Cell Signaling); rabbit polyclonal anti β-tubulin (1:4000, ab6046, abcam), anti-CHD7 (1:500, ab117522, abcam), anti-DUSP4 (1:500, ab72593, abcam) and anti-HA (1:1000, ab9110, abcam); goat polyclonal anti-Lamin B (1:5000, sc-6216, Santa Cruz).

Assay for Transposase-Accessible Chromatin (ATAC)

Intact nuclei were extracted from freshly isolated cells (50,000) of each condition (Ctr, shCHD7, shBMI1, shCHD7;BMI1) and ATAC libraries were produced as previously described (Buenrostro et al., 2013). Three independent biological replicates were used for each condition.

qPCR analyses were performed on a Stratagene Mx3000P thermal cycler (Agilent Technologies) using PrecisionPlus 2X Mastermix (Primerdesign) following the manufacturer's guidelines. The following primer pairs were used:

FW1: GAAGAGGCGGACCCAGCGGT, Rev1: TTCCTGCCGGTCATCTCGCTT (KAT6B);

FW2: GCGAGGAAGAGAAGAGAACCCG, Rev2: GCTCCGACTGCTATGTGACCG (433-259 down);

FW3: AAAGGAAATAGCCGGCTGAGGA, Rev3: AGTCTGGGGCTAGGAGGTGT (404-576 up);

FW4: AGGACTCGCTCGCAGTTTCG, Rev4: AAGGTCTCGGAAGTGGAGGCTC (1270 – 1509 up).

Technical triplicates for each sample were analysed. The Ct values of all experimental primer pairs were normalised against the Ct value of a control locus where DNA accessibility is

known to be constitutively high (KAT6B), and then expressed as a percentage of the control group, with control being 100%.

Gene expression analysis of cell line BMI1 / CHD7 knockdowns

RNA sequencing was performed using the Illumina HiSeq2000 platform with poly(A) enrichment and paired end reads. Two biological replicates of the following conditions were used: Ctrl, shBMI1, shCHD7, shCHD7;shBMI1. Between 40 and 60 million reads were obtained in all cases. Illumina's BaseSpace was used to perform TopHat (Trapnell et al., 2009) alignments of all reads, and to run CuffLinks (Trapnell et al., 2010) for gene expression estimation followed by differential expression analysis. Log2 fold changes between conditions (Ctrl vs shBMI1, Ctrl vs shCHD7 and Ctrl vs shCHD7;shBMI1) were determined and genes differentially expressed in the replicates were determined as having a log2 fold change > +/- 0.5, and those with a p-value < 0.05 were further analysed. The datasets are available in GEO, GSE83696.

Gene expression analysis of BMI1^{High};CHD7^{Low} patients

A z-score for BMI1 and CHD7 gene expression was produced for all patients in the cohort (Cho et al., 2011). Patients were classified as having a BMI1^{High};CHD7^{Low} signature if the BMI1 z-score was >0.2 and if the CHD7 z-score was <-0.2. The mean expression of every gene for patients in the BMI1^{High};CHD7^{Low} group vs the remaining group of patients was determined in order to evaluate differential expression. A permutation test was performed (n=1000) to assign a p-value for the observed difference in expression. Differentially expressed genes were cross-referenced with differentially expressed genes in the shCHD7 cells vs shCHD7;shBMI1 cells. Genes that agreed as being either upregulated or downregulated in both cohorts, and were significant in both cohorts (p<0.05) were shortlisted for further analysis.

Pre-processing gene expression data from microarrays

Microarray data were subjected to the same pre-processing steps in all cases. Raw microarray data were first normalised using the RMA transformation (Irizarry et al., 2003) then the probe set annotation for the appropriate platform was used to assign probe sets to genes. Where multiple probe sets matched a single gene, the maximum value was used.

Pre-processing gene expression data from RNA sequencing

The three primary MB lines considered in this study (ICb1299, ICb1595 and CHLA-01-Med), in addition to the ICb1299 lentiviral knockdown conditions, were characterised by RNA sequencing. The raw data were manually checked with FastQC (Babraham Institute) to ensure that the reads were of sufficient quality and that no adapter sequences were found. The ICb1299 conditions were aligned to the Ensembl human reference genome GRCh38 using HISAT2 (Kim et al., 2015) followed by processing with featureCounts (Liao et al., 2014) to obtain gene counts. In the case of ICb1595 and CHLA-01-Med we used STAR (Dobin et al., 2013) to align to the same reference genome and obtain gene counts simultaneously.

Principal component analysis

Fig 3A shows the result of applying principal component analysis (PCA) to a published microarray dataset comprising 73 MB samples that have previously been classified by subgroup (Robinson et al., 2012) and the three cell lines described in this study (ICb1299, ICb1595, CHLA-01-Med).

To facilitate a consistent comparison across different gene expression measurement platforms, we first applied the YuGene transformation (Le Cao et al., 2014) to the Robinson microarray data. We then computed the PCA transformation, retaining the top 3 components. Subgroup centroids in the three-dimensional PCA coordinate system were

calculated by assuming that the data follow a multivariate Gaussian mixture model and computing the ellipsoid enclosing 99% of the density.

We applied the same PCA transformation to YuGene-transformed gene count data obtained from RNA sequencing performed on the three MB cell lines included in this study. The ICb1595 sample is readily identifiable as belonging to G3. ICb1299 and CHLA-01-Med both fall within the boundary region between subgroups G3 and G4.

Unsupervised hierarchical clustering

As an additional verification of the molecular identity of the lines ICb1299 and ICb1595 we performed unsupervised hierarchical clustering of the tumour tissue and early passage gene expression data from these samples (Zhao et al., 2012) together with the Robinson cohort (Robinson et al., 2012) and the ICb1299, ICb1595 and CHLA-01-Med cell lines from this study (Fig. S3A). The Robinson and Zhao microarray data were prepared as previously described. Following the addition of a small offset value of 10^{-12} to the TPM values of the RNASeq-derived gene counts, the values were log2 transformed. Clustering was performed using the 100 genes identified by Northcott et al. as being highly differentially expressed between medulloblastoma subgroups (Northcott et al., 2012).

Gene expression heatmaps

Fig. S3C shows gene expression data from the knockdowns in the current study and nine healthy adult cerebellum samples, obtained from the Allen Human Brain Atlas (AHBA) (Hawrylycz et al., 2012). Following the addition of a small offset value of 10^{-12} to the TPM values, both datasets were log2 transformed. The data were standardised by subtracting the AHBA sample mean and dividing by the AHBA sample standard deviation for each gene. We

display gene expression values corresponding to 50 genes previously implicated in medulloblastoma G3 and G4 membership (Northcott et al., 2012).

References

Brett, B. T., Berquam-Vrieze, K. E., Nannapaneni, K., Huang, J., Scheetz, T. E., and Dupuy, A. J. (2011). Novel molecular and computational methods improve the accuracy of insertion site analysis in Sleeping Beauty-induced tumors. PloS one *6*, e24668.

Buenrostro, J. D., Giresi, P. G., Zaba, L. C., Chang, H. Y., and Greenleaf, W. J. (2013). Transposition of native chromatin for fast and sensitive epigenomic profiling of open chromatin, DNA-binding proteins and nucleosome position. Nature methods *10*, 1213-1218.

Cho, Y. J., Tsherniak, A., Tamayo, P., Santagata, S., Ligon, A., Greulich, H., Berhoukim, R., Amani, V., Goumnerova, L., Eberhart, C. G.*, et al.* (2011). Integrative genomic analysis of medulloblastoma identifies a molecular subgroup that drives poor clinical outcome. J Clin Oncol *29*, 1424-1430.

Dobin, A., Davis, C. A., Schlesinger, F., Drenkow, J., Zaleski, C., Jha, S., Batut, P., Chaisson, M., and Gingeras, T. R. (2013). STAR: ultrafast universal RNA-seq aligner. Bioinformatics (Oxford, England) *29*, 15-21.

Hawrylycz, M. J., Lein, E. S., Guillozet-Bongaarts, A. L., Shen, E. H., Ng, L., Miller, J. A., van de Lagemaat, L. N., Smith, K. A., Ebbert, A., Riley, Z. L.*, et al.* (2012). An anatomically comprehensive atlas of the adult human brain transcriptome. Nature *489*, 391-399.

Irizarry, R. A., Hobbs, B., Collin, F., Beazer-Barclay, Y. D., Antonellis, K. J., Scherf, U., and Speed, T. P. (2003). Exploration, normalization, and summaries of high density oligonucleotide array probe level data. Biostatistics *4*, 249-264.

Khodosevich, K., Inta, D., Seeburg, P. H., and Monyer, H. (2007). Gene expression analysis of in vivo fluorescent cells. PloS one *2*, e1151.

Kim, D., Langmead, B., and Salzberg, S. L. (2015). HISAT: a fast spliced aligner with low memory requirements. Nature methods *12*, 357-360.

Le Cao, K. A., Rohart, F., McHugh, L., Korn, O., and Wells, C. A. (2014). YuGene: a simple approach to scale gene expression data derived from different platforms for integrated analyses. Genomics *103*, 239-251.

Liao, Y., Smyth, G. K., and Shi, W. (2014). featureCounts: an efficient general purpose program for assigning sequence reads to genomic features. Bioinformatics (Oxford, England) *30*, 923-930.

Merve, A., Dubuc, A. M., Zhang, X., Remke, M., Baxter, P. A., Li, X. N., Taylor, M. D., and Marino, S. (2014). Polycomb group gene BMI1 controls invasion of medulloblastoma cells and inhibits BMP-regulated cell adhesion. Acta neuropathologica communications *2*, 10.

Morrissy, A. S., Garzia, L., Shih, D. J., Zuyderduyn, S., Huang, X., Skowron, P., Remke, M., Cavalli, F. M., Ramaswamy, V., Lindsay, P. E.*, et al.* (2016). Divergent clonal selection dominates medulloblastoma at recurrence. Nature *529*, 351-357.

Northcott, P. A., Shih, D. J., Peacock, J., Garzia, L., Morrissy, A. S., Zichner, T., Stutz, A. M., Korshunov, A., Reimand, J., Schumacher, S. E.*, et al.* (2012). Subgroup-specific structural variation across 1,000 medulloblastoma genomes. Nature *488*, 49-56.

Robinson, G., Parker, M., Kranenburg, T. A., Lu, C., Chen, X., Ding, L., Phoenix, T. N., Hedlund, E., Wei, L., Zhu, X.*, et al.* (2012). Novel mutations target distinct subgroups of medulloblastoma. Nature *488*, 43-48.

Trapnell, C., Pachter, L., and Salzberg, S. L. (2009). TopHat: discovering splice junctions with RNA-Seq. Bioinformatics (Oxford, England) *25*, 1105-1111.

Trapnell, C., Williams, B. A., Pertea, G., Mortazavi, A., Kwan, G., van Baren, M. J., Salzberg, S. L., Wold, B. J., and Pachter, L. (2010). Transcript assembly and quantification by RNA-Seq reveals

unannotated transcripts and isoform switching during cell differentiation. Nature biotechnology *28*, 511-515.
Zhao, X., Liu, Z., Yu, L., Zhang, Y., Baxter, P., Voicu, H., Gurusiddappa, S., Luan, J., Su, J. M., Leung, H. C., and Li, X. N. (2012). Global gene expression profiling confirms the molecular fidelity of primary tumor-based orthotopic xenograft mouse models of medulloblastoma. Neuro-oncology *14*, 574-583.
